# Age-related anabolic resistance and post-absorptive muscle protein synthesis: integrative evidence from a systematic review and meta-analysis

**DOI:** 10.3389/fphys.2026.1740284

**Published:** 2026-06-05

**Authors:** Jonas Boritz Kristiansen, Kristian Vissing, Jakob Lindberg Nielsen

**Affiliations:** 1 Exercise Biology, Department of Public Health, Aarhus University, Aarhus, Denmark; 2 Department of Geriatric Medicine, Odense University Hospital, Odense, Denmark; 3 Department of Sports Science and Clinical Biomechanics, University of Southern Denmark, Odense, Denmark

**Keywords:** aging, muscle protein synthesis, muscle protein breakdown, amino acid metabolism, anabolic resistance, exercise, postprandial metabolism, stable isotope tracers

## Abstract

**Introduction:**

Age-related declines in skeletal muscle mass and function are partly attributed to anabolic resistance, a diminished stimulation of muscle protein synthesis (MPS) following nutrient intake or exercise. However, the magnitude and physiological specificity of this phenomenon remain incompletely defined. This systematic review and meta-analysis examined age-related differences in MPS across post-absorptive, post-prandial, post-exercise, and combined post-prandial/-exercise states in healthy adults.

**Methods:**

A systematic literature search was conducted in November 2025 across major databases. Eligible studies compared MPS between young (18–35 years) and older (≥60 years) adults using stable isotope tracer techniques under controlled conditions. Random-effects meta-analyses were conducted for post-absorptive and post-prandial conditions, while post-exercise and combined post-prandial/-exercise findings were synthesized qualitatively due to substantial methodological heterogeneity. Meta-regression explored potential moderators, and risk of bias was assessed using the ROBINS-I tool.

**Results:**

Forty-six studies including 1,280 participants (700 older adults, 580 young) met the inclusion criteria. Post-absorptive MPS was significantly lower in older compared with younger adults (SMD = −0.167; 95% CI: −0.313 to −0.021; p = 0.025; I² = 18.4%). Post-prandial MPS was also reduced in older adults (SMD = −0.348; 95% CI: −0.627 to −0.070; p = 0.014; I² = 83.4%), with consistent directionality across sensitivity analyses. Post-exercise (5/8) and combined post-prandial/-exercise (7/9) studies reported no significant age-related difference. Overall risk of bias was low to moderate.

**Discussion:**

These findings demonstrate modest but significant reductions in both post-absorptive and post-prandial MPS with aging, consistent with basal and nutrient-related anabolic resistance. However, the preserved MPS response to exercise suggests maintained anabolic potential when appropriate stimuli are provided. Evidence for combined post-exercise MPS remains mixed, with evidence leaning toward a similar response in young and older adults. Standardization of tracer protocols and sex-specific analyses are warranted to refine mechanistic understanding of age-related anabolic heterogeneity.

## Introduction

Skeletal muscle mass is a strong predictor of longevity and physical function in humans (Visser et al., 2025; Zhou et al., 2023). Conversely, conditions such as disuse, aging and chronic disease are associated with the progressive loss of muscle mass, muscle quality and muscle strength, ultimately culminating in sarcopenia - a state characterized by physiological and functional decline (Cruz-Jentoft et al., 2019). Muscle mass may be maintained, increase or decrease depending on the dynamic balance between muscle protein synthesis (MPS) and muscle protein breakdown (MPB) (Hodson et al., 2019). To this end, a positive net change in muscle mass in response to both dietary stimuli and exercise stimuli is generally believed to be primarily driven by an increase in MPS (Morton et al., 2015, Morton et al., 2018b), while reduced MPS rate rather than increased MPB appears to be the main driver of negative net changes in muscle mass in healthy aged individuals (Brook et al., 2022; Morton et al., 2018a; Phillips et al., 2009; Wilkinson et al., 2018). This age-related blunting of MPS responsiveness to anabolic stimuli, termed anabolic resistance, is considered a central mechanism underlying the development of sarcopenia (Morton et al., 2018b), emphasizing the importance of understanding how MPS regulation is altered with aging.

The regulation of MPS is largely governed by nutrient- and exercise-sensitive intracellular signaling pathways. The mechanistic target of rapamycin (mTOR) serves as the more direct nodal point that integrates anabolic cues to regulate translational efficiency and capacity (Roberts et al., 2023). In accordance, essential amino acids (EAAs), particularly leucine, and exercise, specifically resistance exercise (RE) through both intrinsic and systemic mechanisms, are considered potent activators of mTOR for MPS (Bar-Peled and Sabatini, 2014; Roberts et al., 2023).

Anabolic resistance refers to an attenuated MPS and anabolic signaling response to RE and/or amino acid ingestion (Morton et al., 2018b). It is suggested to be increasingly evident with advancing age, yet its underlying basis appears multifactorial. Some human studies report modest declines in basal, post-absorptive MPS rates with aging (Groen et al., 2016; Guillet et al., 2004), while others report reduced MPS rates accompanied by reduced mTORC1 signaling following amino acid intake or RE (Cuthbertson et al., 2005; Fry et al., 2011). However, not all studies report diminished MPS rates. Some have reported comparable levels of MPS rates in the post-absorptive state (Smeuninx et al., 2017) and similar responses to amino acids (Chevalier et al., 2011) and RE (Brook et al., 2016). Moreover, combining amino acid ingestion with RE elicits early increases of MPS in young individuals (1–3h post-exercise), but delayed increases in older individuals (3-6h post-exercise), suggesting a temporal shift of the anabolic response with aging (Drummond et al., 2008). These discrepancies underscore the complexity of anabolic resistance and highlight a need for systematic evaluation to disentangle methodological, physiological and context-specific influences.

The present review specifically aims to evaluate the acute anabolic responsiveness of skeletal muscle to the post-absorptive state as well as nutritional intake and/or exercise in younger versus older adults. To achieve this, we delimit the review to studies that have assessed short-term outcomes such as primarily MPS, but to a lesser extent also amino acid kinetics, in fasting condition and in response to protein ingestion, and/or an acute bout of exercise under controlled conditions. On the other hand, we exclude prolonged intervention studies (e.g., longitudinal resistance training), as such prolonged settings are typically not as strictly standardized. Specifically, although some of these types of studies provide protein supplementation and occasionally monitor habitual diet, their inherent heterogeneity in training protocols, compliance, and background diet makes them immediately less suitable for isolating the mechanistic concept of age-related anabolic resistance.

## Design and methods

### Search strategy

A systematic literature search was performed in Ovid MEDLINE and Ovid EMBASE on July 17, 2025 (updated 01/11/2025), as these databases are considered to comprehensively cover journals in health and clinical sciences. The search strategy addressed (1) the primary objective of age-related skeletal muscle protein synthesis.

**Figure 1:**
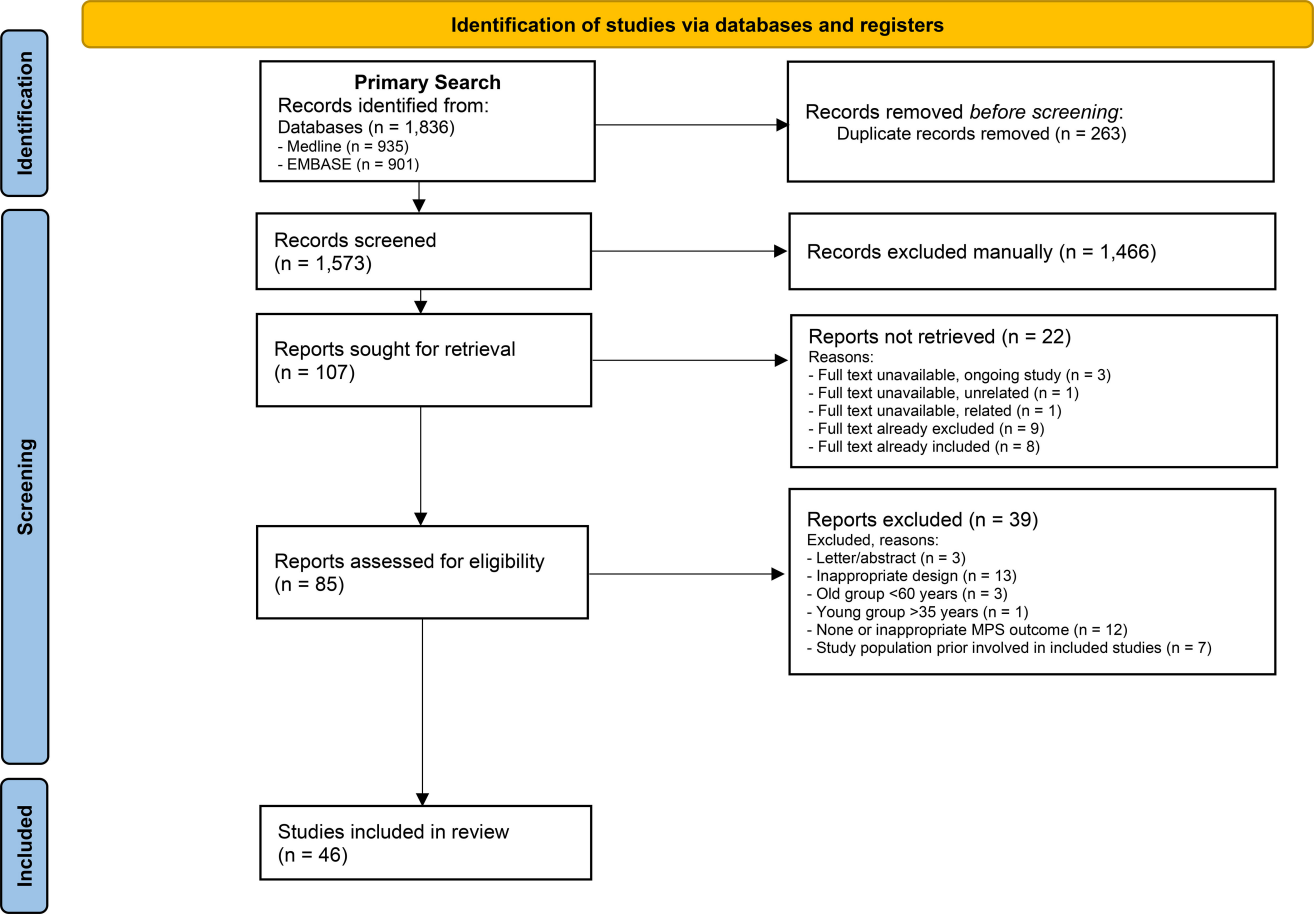
Flow chart. A total of 1836 records were identified through a primary database search (Medline and EMBASE). After removal of 263 duplicates and 1466 manually excluded records, 107 reports were sought for retrieval. 22 reports were not retrieved, and 39 reports were excluded after full-text screening. In total, 46 studies met the inclusion criteria and were included in the review. Records were identified through systematic database searches designed to capture studies comparing older and younger adults in relation to skeletal muscle physiology and anabolic responses. Search terms included synonyms and Medical Subject Headings (MeSH) related to age classification (e.g., “older adults,” “elderly,” “younger adults,” “young adult” [MeSH]), muscle tissue (e.g., “muscle*,” “Muscles” [MeSH]), and anabolic resistance or protein synthesis pathways (e.g., “anabolic resistance,” “protein biosynthesis” [MeSH], “muscle protein synthesis”). Age-related terms for older and younger populations, muscle-related terms and anabolic resistance/protein metabolism terms were combined using OR, then linked using AND. Both free-text terms and controlled vocabulary (MeSH) were employed to ensure comprehensive coverage. No date restrictions were applied.

A comprehensive search string was developed encompassing relevant search categories (aging, young and MPS-related terms) (find full search string in **Figure 1**). Relevant Medical Subject Headings (MeSH) terms were incorporated. Boolean operators AND and OR were used to combine terms. No limits were applied regarding publication date, language, or species. Additional studies were identified by screening the reference lists of included papers and related reviews.

### Eligibility criteria

#### Eligible studies

Randomized and non-randomized clinical trials, including parallel-group and comparative studies directly comparing young and older participants using standardized between-group methods, were eligible for inclusion. Non-randomized designs were accepted to allow for age-based group distinction (i.e., young vs. older), as between-group randomization is not feasible in this context. Only studies published in English were considered, with no restrictions on publication date.

#### Eligible populations

Healthy young and older adults of both sexes were allowed. Studies were included if the mean age of participants in the young was between 18 and 35 years and ≥60 years in the older group. These thresholds were chosen based on population data showing that age-related muscle loss, including sarcopenia, typically emerges during the sixth decade compared with younger adults (Suetta et al., 2019; von Haehling et al., 2010). With the aim of reducing potential heterogeneity, only inactive to recreationally active participants were included, while those engaged in strength training were excluded. To specifically assess age-related skeletal muscle protein synthesis, studies involving participants with medical conditions (e.g., diabetes, rheumatic disease, cancer) or the use of medications (e.g., glucocorticoids) known to significantly affect muscle protein turnover were excluded.

#### Eligible study interventions

For the assessment of postabsorptive skeletal muscle protein synthesis, studies without or with an intervention were included. To examine the effects of well-established anabolic stimuli on acute MPS, studies that investigated MPS in response to a single stimulus - either amino acid/protein supplementation and/or exercise - were eligible. A single acute bout of exercise was allowed, provided that exercise intensity, load, and volume were standardized and adequately controlled between study groups; this included resistance and/or endurance exercise (e.g., walking, cycling). Amino acids or protein could be administered orally (liquid or solid) or intravenously. Protein supplementation (type and amount) had to be clearly described to meet eligibility. Co-ingestion of other macronutrients was permitted, as long as intake was standardized between groups. Studies involving co-administration of ergogenic aids (e.g., vitamins, creatine) or pharmaceuticals (e.g., glucocorticoids) with potential to influence age-related differences in the MPS response were excluded.

#### Eligible outcome measures

The primary outcome was the evaluation of MPS in the post-absorptive condition or in response to an acute amino acid/protein and/or exercise stimulus. Eligible studies evaluating an intervention were required to assess MPS within ≤ 24 hours of the intervention, as the response is known to peak within ~24 hours and subsequently return toward baseline within 48 hours post-stimulus (Damas et al., 2015; Phillips et al., 1997).

Only muscle-specific protein synthesis data were included. Consequently, studies reporting whole-body protein synthesis or using indirect methods to estimate MPS (e.g., two- or three-pool arteriovenous balance) were excluded. Accordingly, eligible studies had to report MPS as a fractional synthetic rate derived from the precursor–product method. Subfractions must involve contractile components of skeletal muscle, thus mixed-muscle, myofibrillar, or myosin heavy chain fractions were considered acceptable. Both post-absorptive, post-prandial and post-exercise MPS measures were extracted. If multiple MPS measurements using different methodologies (e.g., tracer, precursor–product, or subfraction approaches) were available, the one yielding greater overall homogeneity was prioritized. When MPS was evaluated in more than one time interval, the time interval with the greatest between-group difference was prioritized.

Secondary outcomes included estimated MPB and plasma amino acid concentrations. MPB was assessed using tracer kinetics calculated using the one-, two- or three-pool models, while plasma amino acids were evaluated using area under the curve (AUC) when available, or the time point showing the greatest between-group difference. Both systemic and muscle-specific estimates of muscle protein breakdown were considered: rate of appearance of amino acids in plasma provides an indirect estimate of protein breakdown by reflecting net amino acid release from muscle into circulation, whereas direct tracer release from muscle offers a direct measure of skeletal muscle protein degradation. Essential amino acids and especially leucine was prioritized for extraction, due to the importance of stimulating MPS (Phillips, 2016).

#### Risk of bias assessment

Risk of bias assessment of MPS studies was performed using the ‘Risk Of Bias In Non-randomized Studies of Interventions (ROBINS-I): detailed guidance’ (Sterne et al., 2016). ROBINS-I evaluate internal validity within the domains: ‘Confounding Bias’, ‘Participant Selection Bias’, ‘Intervention Classification Bias’, ‘Deviation from the Intended Intervention’, ‘Missing Data’, ‘Missing Outcomes’, and ‘Selection of Reported Results’ against a hypothetical target randomized trial and has been shown by the Cochrane to be a conceptually rigorous tool.

### Data collection and analysis

#### Data extraction

Study, participant, methodological, and intervention details were extracted into a customized sheet. Extracted data included: (i) study information (author, year, country, design, conflicts of interest); (i) participant characteristics (age, sex, body weight, health/exercise status); (iii) primary outcome, MPS (tracer type, infusion protocol, precursor/fraction, biopsy site and timing, analytical method, sample size, pre/post statistics); (iv) intervention parameters, protein supplementation (type, amount, timing) and exercise protocols [resistance: load, sets, repetitions, rest, contraction type/duration, timing; endurance: intensity, duration, continuous vs. interval, timing]; and (v) secondary outcomes, protein breakdown and plasma amino acid (methods, sample size, pre/post statistics).

### Method of data synthesis

#### Quantitative vs. qualitative analysis

The choice between a formal meta-analysis and a more qualitative synthesis (e.g., effect direction plots) was guided by the degree of homogeneity (experimental/methodological) across studies. If the eligible study sample, total sample size, and between-study heterogeneity were considered acceptable, a meta-analysis was performed to obtain a pooled estimate of the effect. Conversely, when substantial heterogeneity was detected and could not be explained, such as when effect estimates were not comparable due to major design or methodological differences (e.g., study design, participant characteristics, or tracer methodology), a narrative synthesis with effect direction plots was considered more appropriate.

#### Meta-analysis

All statistical analyses were performed using R Studio (version 4.5.1). The standardized mean difference (SMD) with corresponding standard errors (SE) was used as the primary effect measure. Where necessary, medians were converted to means and interquartile ranges to standard deviations (SD) to ensure comparability across studies. Dissimilar units were recalculated with the aim of homogeneity.

Random-effects models were applied to account for anticipated between-study heterogeneity. Statistical heterogeneity was quantified using Cochran’s Q test and the I² statistic. Publication bias was assessed visually using funnel plots. Sensitivity analyses were performed to examine the robustness of the pooled effect estimate, including leave-one-out analyses. Further, subgroup sensitivity analyses were performed by tracer type (leucine/phenylalanine/other), precursor pool (intracellular vs. plasma amino acid), subfraction (mixed vs. myofibrillar protein synthesis), MPS timing, and intervention specific factors (i.e., exercise volume, protein type/amount), whenever possible. These analyses were intended to explore potential sources of heterogeneity and assess whether specific methodological choices influenced the overall effect estimates.

To evaluate the effect of any intervention (protein supplementation/exercise) on MPS, absolute change scores (post-pre intervention) were used as the primary effect measure. To assess the robustness of the findings, sensitivity analysis was conducted using relative change scores and varying assumptions for the pre–post correlation coefficient (r=0.7) when imputing standard deviations of change.

### Effect direction plots

Effect direction plots were used to visualize the consistency and direction of findings.

## Results

### Literature search

A systematic literature search was conducted on 1st November 2025 (JLN), identifying 1,836 records (see **Figure 1** for details). Following removal of duplicates, title/abstract screening, full-text review (JB/JLN), and cross-checking of topic-specific systematic reviews and reference lists, 46 studies met the inclusion criteria (**Figure 1**).

### Participants

The included studies eligible for analyses (n=46) comprised a total of 1,280 adult participants (older: 700; young: 580). All participants were classified as healthy, with most studies explicitly reporting health criteria (38 of 46). The mean age of the older and younger groups ranged from 64 to 80 years (mean: 70 years), and 20 to 35 years (mean: 26 years), respectively. Physical activity status was infrequently described (15 of 46 studies); when reported, it was typically characterized in broad terms (e.g., “recreationally active”) and only rarely quantified (3 of 15 studies) (**Supplementary Tables S1**–**S4**).

### Study interventions

All results are summarized in **Supplementary Tables S1**–Y**S6**.

Of the 46 eligible studies (Atherton et al., 2017; Babraj et al., 2005; Balagopal et al., 1997; Brook et al., 2016; Chevalier et al., 2011; Cuthbertson et al., 2005; Dillon et al., 2011; Drummond et al., 2008; Durham et al., 2010; Fry et al., 2011; Gorissen et al., 2014; Groen et al., 2016; Guillet et al., 2004; Hasten et al., 2000; Henderson et al., 2009; Hermans et al., 2023; Horwath et al., 2025; Katsanos et al., 2006; Kiskini et al., 2013; Koopman et al., 2006a, Koopman et al., 2009; Kumar et al., 2012, Kumar et al., 2009; Lalia et al., 2017; Lamon et al., 2016; Mayhew et al., 2009; Michie et al., 2024; Mitchell et al., 2017; Paddon-Jones et al., 2004; Pennings et al., 2011; Phillips et al., 2017; Rasmussen et al., 2006; Rooyackers et al., 1996; Sheffield-Moore et al., 2005; Smeuninx et al., 2017; Symons et al., 2011, Symons et al., 2009; Toth et al., 2005; Volpi et al., 2000, Volpi et al., 1999, Volpi et al., 2001; Walrand et al., 2008; Welle et al., 1993, Welle et al., 1995, Welle et al., 1994; Yarasheski et al., 1993), 37 studies evaluated post-absorptive MPS, while 18, 10 and 10 evaluated post-prandial, post-exercise or combined post-exercise/prandial MPS, respectively (Tabel S1-S4). Protein supplementation studies involved various protein sources mostly consisting of casein/whey (n=11), EAA mixture (n=13) or mixed meals (n=3) either provided orally (n=24) or intravenously (n=4) ranging between 2.5 to 90 g occasionally supplemented with 40–60 g carbohydrate. Exercise studies involved resistance (18 of 20) or endurance exercise (2 of 20). RE studies involved 1–2 exercises targeting the MPS-evaluated muscle (VL) with most studies involving 4–10 sets using a loading schema of 70-80% of 1RM. The endurance exercise studies involved continuous exercise (40–45 min) at 40-55% of VO_2_peak. One RE study and two EE studies were excluded from the exercise-related analyses. The RE study (Reitelseder et al., 2021) assessed post-exercise MPS (<24 h) after three consecutive training sessions, and the EE studies (Durham et al., 2010; Sheffield-Moore et al., 2004) represented a very limited evidence base (n = 1 for post-exercise and n = 1 for combined post-exercise/post-prandial), preventing identification of meaningful patterns. Additionally, the exercise-induced MPS response in EE was expected to differ from that observed following RE.

Muscle protein breakdown was evaluated in 13 trials, mostly in response to protein supplementation (n=10) and less frequently to exercise (n=1) and the combination of protein supplementation and exercise (n=2). Amino acid concentration was evaluated in 12 trials evaluating post-prandial MPS.

### Experimental methodology

All results are summarized in **Supplementary Tables S1**–**S6**.

Data extracted included MPS based on the precursor/product method (46 of 46 studies). In most studies, either isotope marked phenylalanine (n=29) or leucine (n=15) was utilized as tracer, with intracellular (n=22) or plasma amino acid/α-ketoisocaproate enrichment (n=23) as the precursor product pool. Sampling from muscle vastus lateralis was performed in all studies (46 of 46) and all studies reporting either mixed (n=33) or myofibrillar (n=13) subfractions. Studies obtaining long-term MPS (>24 hours) were not deemed eligible for analyses (Marshall et al., 2023; Morgan et al., 2024). Studies evaluating MPB, interchangeable utilized the one- (n=7), two- (n=2) or three-pool model (n=4) to estimate muscle protein breakdown.

### Systematic analyses

The decision to perform quantitative meta-analyses was guided by the degree of methodological and clinical homogeneity across studies. Post-absorptive and post-prandial tracer studies exhibited sufficient consistency in participant characteristics, tracer methodology, timing of measurements, and outcome reporting, alongside an adequate number of studies, to permit robust pooling of effect sizes using random-effects meta-analysis. In contrast, post-exercise and post-prandial/exercise conditions were characterized by considerable variability in these parameters, coupled with a limited number of studies. Given this heterogeneity, results were summarized using a structured qualitative synthesis supported by effect direction plots to convey the overall trends.

One study was excluded from the post-absorptive meta-analysis, as it was designed as a within-subject parallel study (non-exercise vs. exercise) without a between-condition wash-out period (Reitelseder et al., 2021), and therefore cross-transfer effect between conditions cannot be ruled out (Hendy and Lamon, 2017). In addition, a number of studies were excluded from the meta-analysis of post-prandial data (Cuthbertson et al., 2005; Kiskini et al., 2013; Koopman et al., 2009; Welle et al., 1994), as they only reported post scores, disabling calculation of change scores.

## Meta-analysis and -regression analyses

### Post-absorptive MPS

**Figure 2:**
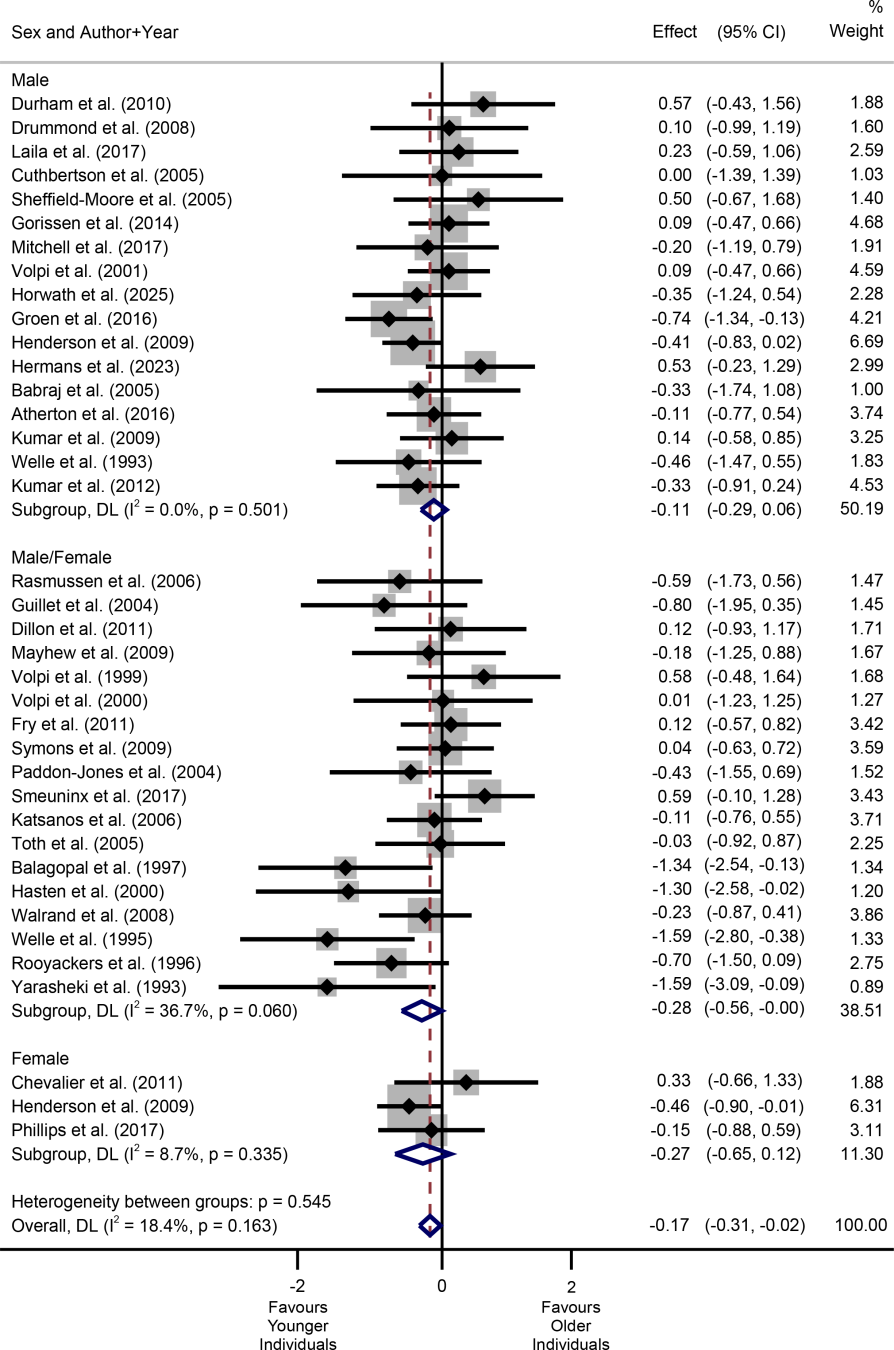
Forest plot, post-absorptive Muscle Protein Synthesis (MPS). Forest plot showing standardized mean differences (SMD) in post-absorptive MPS between older and younger individuals. Each study is represented by a square (effect estimate) and horizontal line (95% CI), with the size of the square reflecting the study weight in the random effects model. Open diamonds represent pooled estimates for each subgroup (male, mixed male/female, and female) and the overall effect.

A meta-analysis of 37 studies demonstrated a small but statistically significant reduction in post-absorptive MPS in older compared with younger adults (SMD = −0.167; 95% CI: −0.313 to −0.021; p = 0.025) (**Figure 2**). Subgroup analyses by sex revealed a significant effect in mixed-sex (male/female) studies (SMD = −0.281; 95% CI: −0.558 to −0.003; p = 0.048), whereas effects were not significant in male-only (SMD = −0.115; 95% CI: −0.292 to 0.062; p = 0.202) or female-only (SMD = −0.266; 95% CI: −0.650 to 0.118; p = 0.175) studies. Heterogeneity was low overall (I² = 18.4%; Q = 45.34, p = 0.163) and within male and female subgroups (I² = 0.0% and 8.7%, respectively), but moderate in mixed-sex studies (I² = 36.7%).

Leave-one-out sensitivity analyses demonstrated that exclusion of any single study did not materially alter the pooled effect estimate (range: –0.15 to –0.20) or its statistical significance. Heterogeneity remained low (I² ≈ 10–21%) and Q-tests were non-significant (p > 0.13), indicating that the overall findings were robust and not driven by any individual study.

Meta-regression analyses revealed that tracer type significantly influenced study-level SMDs (b = 0.37; 95% CI: 0.06–0.69; p = 0.022), with studies using phenylalanine showing higher SMDs than those using leucine. Residual heterogeneity after accounting for tracer type was low (I²_res = 9.9%). No significant association was observed for precursor pool (intracellular vs. plasma; β = 0.24; 95% CI: −0.05 to 0.54; *p* = 0.098) or muscle subfraction (mixed vs. myofibrillar; β = −0.01; 95% CI: −0.36 to 0.33; *p* = 0.934).

There was a trend toward a negative association between MPS duration and study-level SMDs (b = −0.043; 95% CI: −0.091 to 0.004; p = 0.073), suggesting attenuated standardized effects with longer measurement periods. Importantly, infusion duration prior to MPS measurement was significantly associated with study-level SMDs (b = −0.11; 95% CI: −0.21 to −0.01; p = 0.029), indicating that longer pre-labeling periods were related with smaller SMDs.

### Post-prandial MPS

**Figure 3:**
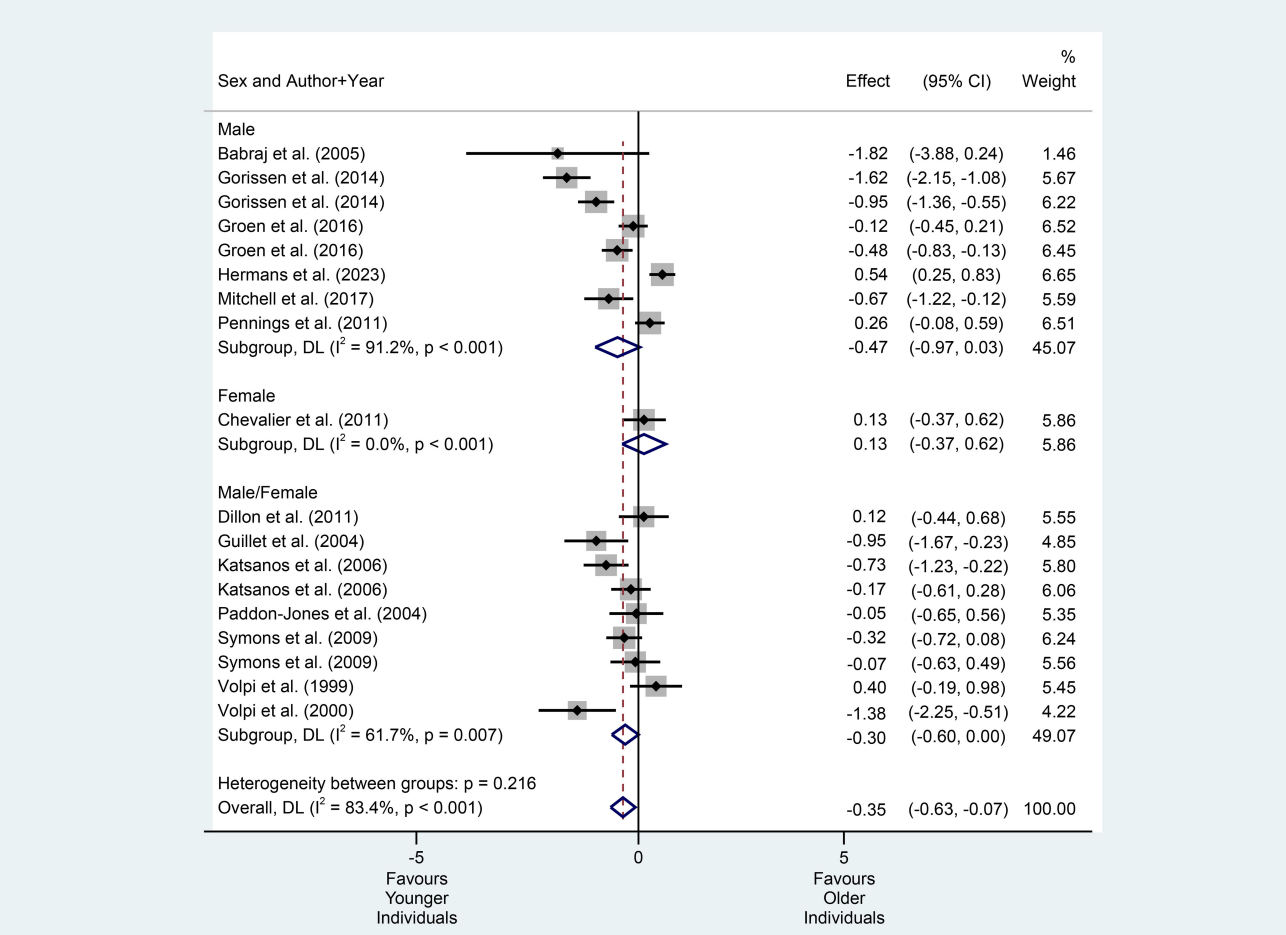
Forest plot, post-prandial Muscle Protein Synthesis (MPS). Forest plot showing standardized mean differences (SMD) in post-prandial MPS between older and younger individuals. Each study is represented by a square (effect estimate) and horizontal line (95% CI), with the size of the square reflecting the study weight in the random effects model. Open diamonds represent pooled estimates for each subgroup (male, female, and mixed male/female) and the overall effect.

A separate meta-analysis of 14 studies revealed a small-to-moderate pooled effect favoring younger individuals in the post-prandial state (SMD = −0.348; 95% CI: −0.627 to −0.070; *p* = 0.014), with substantial heterogeneity (I² = 83.4%) (**Figure 3**). Subgroup analyses suggested a more negative effect in males (SMD = −0.471; 95% CI: −0.974 to 0.033) compared with females (SMD = 0.125; 95% CI: −0.367 to 0.617), though these subgroup differences were not statistically significant (Q_between = 3.06; *p* = 0.216). Mixed-sex studies showed an SMD of −0.299 (95% CI: −0.600 to 0.003).

Leave-one-out sensitivity analyses demonstrated no undue influence of individual studies on the pooled effect (SMD range: −0.245 to −0.400). Excluding one study rendered the overall effect borderline non-significant (*p* = 0.065), indicating moderate but not critical sensitivity. Additional sensitivity analyses assuming different pre–post correlation coefficients (r = 0.6, 0.7, 0.8) yielded consistent pooled estimates (SMD range: −0.32 to −0.39, all *p* < 0.05), supporting the robustness of the findings. Sensitivity analyses restricted to early-phase MPS measurements resulted in a pooled SMD of−0.354 (95% CI: −0.632 to −0.076; p = 0.013), whereas inclusion of late (± middle) phase measurements attenuated the pooled effect (SMD range: −0.265 to −0.227) but remained statistically significant or borderline significant (p = 0.026–0.050).

Meta-regression analyses were conducted to explore potential sources of heterogeneity. Tracer type (phenylalanine vs. leucine), precursor pool (intracellular vs. plasma), subfraction (mixed vs. myofibrillar), route of administration (oral vs. intravenous), and protein dose were not significantly associated with study-level SMDs (all p > 0.05). The regression coefficients for these moderators were 0.93, −0.37, 0.68, −0.18, and 0.005, respectively. The proportion of between-study variance explained was low to modest (adjusted R²: −16% to 28%), and residual heterogeneity remained moderate (I²_res 30–38%).

### Assessment of publication bias

Visual inspection of the funnel plots (**Supplementary Figures 1, 2**) for the 37 post-absorptive and 14 post-prandial studies indicated that most studies were symmetrically distributed around the pooled SMD, with a few small studies showing more extreme effect sizes. Overall, the plots suggested a low risk of publication bias.

## Effect direction plots

### Post-exercise

**Table 1** illustrates effect directions of post-exercise MPS. 3 of 8 studies reported greater post-exercise MPS in young compared with older individuals, while the remaining 5 out of 8 studies reported no difference between age groups.

**Table 1:** Effect directions plot, post-exercise muscle protein synthesis.

Reference	Study design	Muscle Protein Synthetic Response		
		Young	Neutral	Old
Brook et al. (2016)	NR-PGD		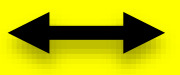	
Fry et al. (2011)	NR-PGD	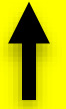		
Kumar et al. (2009)	NR-PGD, AGR	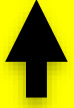		
Kumar et al. (2012)	NR-PGD, AGR, CO	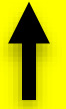		
Lamon et al. (2016)	NR-PGD, AGR, CO		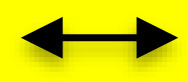	
Sheffield-Moore et al. (2005)	NR-PGD		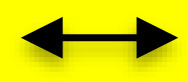	
Michie et al. (2024)	NR-PGD		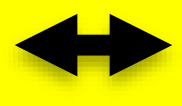	
Mayhew et al. (2009)	NR-PGD		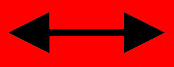	

Effect direction plot summarizing study-level findings for Post-Resistance Exercise MPS. The plot shows the consistency of direction across studies. Effect direction: upward arrows = effect in favor of Young or Old and horizontal arrows= no clear change. Study sample size is reflected by the thickness of the arrows (small/moderate/large thickness: n = ≤8/9-16/>16) pr. age groups. The degree of Risk of Bias is denoted by color shading (yellow: moderate, red: high). Study design: Non-randomized parallel group design (NR-PGD), age-group randomization (AGR), cross-over (CO).

### Post-prandial/-exercise

**Table 2** illustrates effect directions of post-prandial/-exercise MPS. 2 out of 9 studies reported a greater post-prandial/-exercise MPS response in young compared with older individuals, while the remaining 7 out of 9 studies reported no difference between age groups.

**Table 2:** Effect directions plot, post- prandial/-resistance exercise muscle protein synthesis.

Reference	Study design	Muscle Protein Synthetic Response		
		Young	Neutral	Old
Atherton et al. (2017)	NR-PGD, AGR		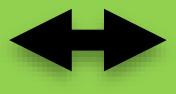	
Hermans et al. (2023)	NR-PGD		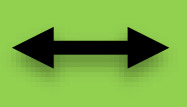	
Pennings et al. (2011)	NR-PGD		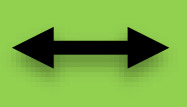	
Drummond et al. (2008)	NR-PGD	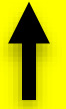		
Horwath et al. (2024)	NR-PGD		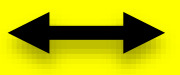	
Koopman et al. (2006a)	NR-PGD, AGR, CO	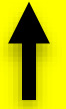		
Laila et al. (2017)	NR-PGD		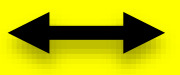	
Phillips et al. (2017)	NR-PGD		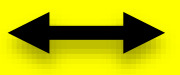	
Symons et al. (2011)	NR-PGD		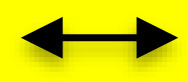	

Effect direction plot summarizing study-level findings for the combined effect of Post-Prandial and Post-Exercise MPS. The plot shows the consistency of direction across studies. Effect direction: upward arrows = effect in favor of Young or Old and horizontal arrows = no clear change. Study sample size is reflected by the thickness of the arrows (small/moderate/large thickness: n = ≤8/9-16/>16) pr. age groups. The degree of Risk of Bias is denoted by color shading (green: low, yellow: moderate). Study design: Non-randomized parallel group design (NR-PGD), age-group randomization (AGR), cross-over (CO).

### Muscle protein breakdown

**Table 3** illustrates effect directions of MPB outcomes from 10 post-prandial studies, 1 post-exercise studies, and 2 post-prandial/-exercise studies. 2 out of 10 studies reported greater post-prandial MPB in young, and 1 out of 10 studies reported greater MPB in old, while the remaining 7 studies reported no difference between age groups. 1 out of 1 study reported no difference in post-exercise MPB. Finally, 1 out of 2 studies reported greater post-prandial/-exercise MPS in young compared to old individuals, while the remaining study reported no difference between age groups.

**Table 3:** Effect directions plot, muscle protein breakdown.

Reference	Study design	Muscle Protein Breakdown		
		Young	Neutral	Old
Protein studies (n=10)				
Gorissen et al. (2014)	NR-PGD, AGR		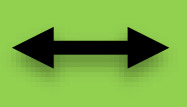	
Groen et al. (2016)	NR-PGD	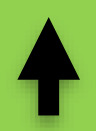		
Pennings et al. (2011)	NR-PGD		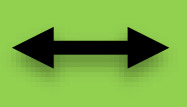	
Chevalier et al. (2011)	NR-PGD			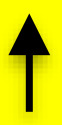
Dillon et al. (2011)	NR-PGD		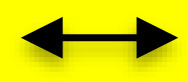	
Katsanos et al. (2006)	NR-PGD, AGR		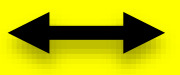	
Koopman et al. (2009)	NR-PGD	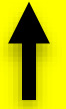		
Paddon-Jones et al. (2004)	NR-PGD		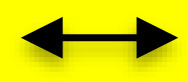	
Volpi et al. (1999)	NR-PGD		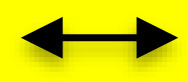	
Volpi et al. (2000)	NR-PGD		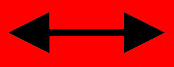	
Exercise studies (n=1)				
Sheffield-Moore et al. (2005)	NR-PGD		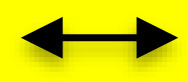	
Combination of exercise and protein studies (n=2)				
Pennings et al. (2011)	NR-PGD		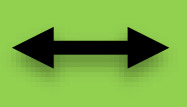	
Koopman et al. (2006a)	NR-PGD	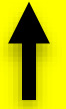		

Effect direction plot summarizing study-level findings for Post-Prandial and/or Post-Resistance Exercise MPB. The plot shows the consistency of direction across studies. Effect direction: upward arrows = effect in favor of Young or Old and horizontal arrows = no clear change. Study sample size is reflected by the thickness of the arrows (small/moderate/large thickness n = ≤8/9-16/>16) pr. age groups. The degree of Risk of Bias is denoted by color shading (green: low, yellow: moderate, red: high). Study design: Non-randomized parallel group design (NR-PGD), age-group randomization (AGR).

### Post-prandial plasma amino acid concentrations

**Table 4** illustrates effect directions of post-prandial plasma amino acid concentrations. 6 out of 12 studies reported greater plasma amino acid concentrations in old compared to young, and 1 out of 12 studies reported greater concentrations in young compared to old. The remaining 5 out of 12 studies reported no difference between age groups.

**Table 4:** Effect directions plot, post-prandial plasma amino acid concentration.

Reference	Study design	Plasma Amino Acids		
		Young	Neutral	Old
Gorissen et al. (2014)	NR-PGD, AGR		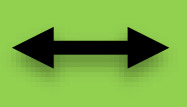	
Groen et al. (2016)	NR-PGD			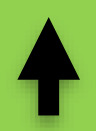
Hermans et al. (2023)	NR-PGD			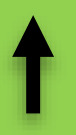
Pennings et al. (2011)	NR-PGD			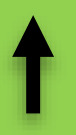
Cuthbertson et al. (2005)	NR-PGD			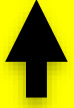
Katsanos et al. (2006)	NR-PGD, AGR		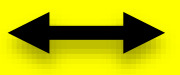	
Kiskini et al. (2013)	NR-PGD	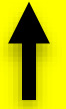		
Koopman et al. (2009)	NR-PGD			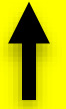
Mitchell et al. (2017)	NR-PGD, NR-SG			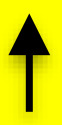
Volpi et al. (1999)	NR-PGD		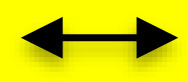	
Welle et al. (1994)	NR-PGD		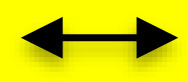	
Volpi et al. (2000)	NR-PGD		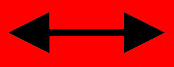	

Effect direction plot summarizing study-level findings for Post-Prandial Plasma Amino Acid Concentration. The plot shows the consistency of direction across studies. Effect direction: upward arrows = effect in favor of Young or Old and horizontal arrows = no clear change. Study sample size is reflected by the thickness of the arrows (small/moderate/large thickness: n = ≤8/9-16/>16) pr. age groups. The degree of Risk of Bias is denoted by color shading (green: low, yellow: moderate, red: high). Study design: Non-randomized parallel group design (NR-PGD), age-group randomization (AGR), non-randomized sub-groups (NR-SG).

## Risk of bias

Risk of Bias were evaluated using the ROBINS-I tool across all studies included (**Supplementary Table S7**). Overall, 8 out of 46 studies were rated as having low risk of bias, 32 studies as moderate risk of bias, and 6 studies as serious risk of bias. The predominant sources of bias were confounding, participant selection, and missing data. Confounding was mainly related to lack of information regarding participant characterization such as habitual diet and physical activity, sex distribution between age groups, as well as insufficient control of pre-study physical activity or nutritional status. Selection bias typically reflected incomplete recruitment details, while missing data arose from unreported exclusion or unclear dropout information.

## Discussion

Despite the abundance of primary data, only limited attempts have been made to systematically synthesize findings comparing MPS responses to anabolic stimuli in young versus old adults. The aim of this review was to systematically evaluate whether postabsorptive MPS and the MPS response to resistance exercise (RE), amino acid/protein intake, and their combination are attenuated in older compared with younger individuals.

To this end, meta-analyses of the current review revealed low to moderate negative effects of increasing age on post-absorptive and post-prandial MPS. On the other hand, for MPS responses to exercise, our systematic evaluation revealed contrasting findings with 3 out of 8 studies reporting lower age-related MPS values. Moreover, when exercise was combined with protein or amino acid feeding, the MPS response remained intact with age in 7 out of 9 studies. Thus, the present findings provide partial support for age-related anabolic resistance.

### Effect of age per se on post-absorptive protein synthesis

The overall meta-analysis demonstrated a modest but significant negative effect of age on muscle protein synthesis, reflecting lower rates in older versus younger adults. To the best of our knowledge, the present data are the first to systematically document reduced MPS in skeletal muscle from aging individuals in the post-absorptive state. Previous experimental work has suggested that net protein balance is primarily driven by attenuating MPS rather than accentuating muscle protein breakdown (Brook et al., 2022; Morton et al., 2018a; Phillips et al., 2009; Wilkinson et al., 2018). Consistent with this, out extracted MPB data (**Table 3**) indicate that breakdown rates are largely preserved with age, with most studies reporting no difference between young and old individuals across post-prandial, post-exercise and combined conditions. Only a minority of studies found greater MPB in either age group, suggesting that impaired MPS, rather than increased MPB, underlies the negative net balance with aging. In line with this, the findings of the present systematic approach support the contention that age-related decline in post-absorptive MPS per se may contribute to the loss of muscle mass observed with aging, i.e., sarcopenia (Cruz-Jentoft et al., 2019), further emphasizing the importance of anabolic stimulating strategies in older adults (Wilkinson et al., 2018).

In the analysis stratification for sex, a small negative effect of age on muscle protein synthesis was observed across both males and females but only reached statistical significance in studies including both sexes. Effects in males and females alone were not significant, probably due to higher heterogeneity in results (for males) and/or less statistical power (for females). Interestingly, some studies have reported higher post-absorptive MPS rates in older women compared to men, which has been suggested to be related to age-related hormonal changes (Henderson et al., 2009; Smith et al., 2012).

Notably, our meta-regression analysis indicated that tracer type significantly influenced post-absorptive MPS, with studies using phenylalanine tracers reported to exhibit higher or near-zero SMDs compared with leucine tracers. These results align with prior evidence indicating leucine as an independent stimulator of MPS (Holwerda et al., 2019; Zaromskyte et al., 2021) and a greater sensitivity to leucine-mediated MPS in younger adults compared with the elderly (Katsanos et al., 2006). Thus, the present data may point towards leucine as a significant factor in age-related anabolic resistance. Furthermore, there was a trend toward a greater difference in MPS between young and older participants with longer MPS measurement periods and a significant association with longer pre-MPS infusion durations. These data suggest that extended duration of potential tracer incorporation may accentuate the apparent anabolic advantage of younger muscle.

### Effect of age per se on post-prandial protein synthesis

The pooled analysis of studies evaluating post-prandial MPS demonstrated a small-to-moderate effect favoring younger individuals, suggesting an attenuated anabolic response to protein supplementation with advancing age. These data support the concept of age-related anabolic resistance.

The present data add quantitative support to previous qualitative observations (Shad et al., 2016), who reported mixed evidence for post-prandial anabolic resistance in older adults (8 study arms supporting anabolic resistance and 13 reporting no effect). By synthesizing findings across trials, our results clarify this discrepancy and suggest that, overall, aging is associated with a modest but measurable attenuation of the MPS response following protein ingestion.

Although the heterogeneity in the analysis was quite substantial, our sensitivity analyses supported the robustness of the age-related attenuation in post-prandial MPS. Specifically, sensitivity analyses stratified by MPS timing did not materially alter the overall conclusions of the post-prandial MPS analysis. However, these timing-specific analyses were based on only three studies reporting multiple post-prandial MPS time points and should therefore be interpreted with caution. When stratifying for sex, indications of a more pronounced effect in males compared with females was observed. Limited power or sex-specific variability in postprandial muscle protein synthesis may explain why these differences did not reach statistical significance. The lack of significant associations in the meta-regression suggests that methodological factors such as tracer type, precursor pool, or protein dose are unlikely to explain a substantial part of the observed between-study heterogeneity. Rather, this pattern suggests that biological factors may largely determine age-related differences in the anabolic response.

Potential contributors to this age-related reduction in post-prandial MPS may include reduced amino acid appearance in the circulation due to slower gastric emptying and intestinal absorption (Chapman et al., 2021). It could also relate to, diminished delivery of amino acids to skeletal muscle due to reduced muscle blood-flow and microvascular perfusion (Banks et al., 2022), and/or decreased capacity of amino acid uptake from the bloodstream into muscle tissue. In addition, intrinsic myocellular factors, such as blunted activation of the mTORC1 signaling pathway and reduced sensitivity to amino acids including leucine (Cuthbertson et al., 2005; Katsanos et al., 2005, Katsanos et al., 2006), may further limit the stimulation of muscle protein synthesis with advancing age.

The present data indicate similar or higher plasma amino acid (leucine, EAA) availability in aging adults (**Table 4**) consistent with recent findings in response to normal and high protein meals (Herpich et al., 2025), however peak amino acid concentrations seems delayed with age (Herpich et al., 2025; Milan et al., 2015). Together these data suggest that the age-related impairment of post-prandial MPS may be governed by age-related differences in local delivery, uptake or myocellular amino acid sensitivity, rather than bioavailability of amino acids. Lastly, older adults may possess dissimilar sensitivity towards amino acid–stimulated MPS. However, this notion is largely based on indirect evidence. Cuthbertson et al. (2005) found that MPS responses to protein supplementation were similar between young and older adults at doses ≤5 g, and higher in young adults at doses ≥10 g, despite the elderly consistently exhibiting higher plasma leucine concentrations. Furthermore, MPS levels of older adults seem to plateau at lower absolute MPS levels despite increasing protein doses and plasma leucine concentrations, suggesting a lower capacity for amino acid uptake and/or MPS capacity (Cuthbertson et al., 2005). These data coincide with superior mTOR and p70^S6K^ phosphorylation response to 10 g of protein supplementation in young compared to older adults (Cuthbertson et al., 2005). On a related note, elderly showed lower MPS in response to EEA supplementation with 26% leucine (1.7 g) content compared to young, but similar MPS in response to 41% leucine (2.8 g) supplementation (Katsanos et al., 2006). Interestingly, Moore et al. (2015) conducted retrospective analyses (n, old/young = 43/65) indicating that MPS in older adults plateaus at approximately 0.4 g/kg bodyweight (range: 0.21–0.59), whereas in younger adults MPS plateaus at around 0.24 g/kg bodyweight (range: 0.18–0.30).

### Effect of age per se on exercise-induced protein synthesis

Based on our extracted studies, evidence suggests conflicting results, however overall leaning to no age-related anabolic resistance to RE, as 3 of 8 studies demonstrated a blunted stimulation of MPS in older compared with younger individuals following acute RE. These findings are overall consistent with a prior systematic review (Shad et al., 2016) that reported 8 study arms supporting anabolic resistance and 9 showing no clear age-related impairment. When viewed collectively, the emerging literature - including the present analysis - indicates that despite heterogeneous results, the balance of evidence does not support the existence of RE–induced anabolic resistance in older adults.

Importantly, the timing of post-exercise MPS assessment may affect our overall conclusions, as the temporal pattern of MPS may differ between age groups. However, studies evaluating temporal patterns are sparse and show inconsistent evidence, with one study reporting a greater early response in young individuals (Kumar et al., 2009), while another showed superior responses in young across both early and late time points (Fry et al., 2011). Overall, longer-term assessments generally suggest a greater cumulative MPS response in young compared with older adults (Brook et al., 2016; Fry et al., 2011), although one study did not support such an age-related difference (Mayhew et al., 2009).

In addition, it is relevant to evaluate responses to life-long high activity levels. Unfortunately, data in highly trained master athletes are very limited. Yet it is noteworthy that, available evidence suggests that age-related attenuation of exercise-induced MPS seems present in highly endurance trained individuals with favorable metabolic health (Doering et al., 2016; McKendry et al., 2019), indicating that aging per se may contribute to altered anabolic responsiveness independent of sedentary behavior.

To this end, two acute tracer studies using classic stable isotope methods consistently report attenuated MPS and mTORC1 signaling during the early recovery period following a single bout of RE (Fry et al., 2011; Kumar et al., 2009). Moreover, one study on longer-term assessment using deuterium oxide confirms that the integrated/cumulative rise in MPS during 3-wks resistance training is reduced in older adults (Brook et al., 2016). However, such attenuation is not a consistent finding and the anabolic response in older muscle may e.g. be influenced by exercise dose, timing, and mode. In accordance, Kumar and coworkers (Kumar et al., 2009) observed age-related differences (young > old) at high loading schemes (60-90% 1RM). Notably, the same authors later demonstrated that increasing the volume of low-load (40% 1RM) RE by doubling the number of sets eliminated the age-related difference in MPS (Kumar et al., 2012), whereas no age-related differences were observed at higher exercise loads (3 or 6 sets at 75% 1RM). This supports a shift in the dose-response relationship, suggesting that higher mechanical loads or training volumes are required to achieve maximal stimulation of MPS with advancing age. Of note, most eligible RE studies evaluating post-exercise MPS applied high-volume protocols (≥ 6 sets at 70% 1RM) targeting the MPS-evaluated muscle group (Brook et al., 2016; Fry et al., 2011; Kumar et al., 2009; Mayhew et al., 2009; Michie et al., 2024; Sheffield-Moore et al., 2005). This amount of RE volume exceeds standard exercise recommendations for older adults (Izquierdo et al., 2021) and may have contributed to the lack of age-related differences in MPS in 5 of the 8 RE studies (Brook et al., 2016; Lamon et al., 2016; Mayhew et al., 2009; Michie et al., 2024; Sheffield-Moore et al., 2005). However, Fry et al. (2011) reported consistently higher MPS in young compared with older adults at all time points following high-volume RE (8 sets at 70%). This may adhere to the study’s large sample size, but it also raises the possibility that younger adults reach an MPS plateau only at higher training volumes. Noteworthy, studies reporting age-related reductions in post-exercise MPS generally included larger sample sizes (≥12 participants) (Fry et al., 2011; Kumar et al., 2012, Kumar et al., 2009), whereas studies showing no difference tended to involve smaller cohorts (≤10 participants) (Brook et al., 2016; Lamon et al., 2016; Mayhew et al., 2009; Sheffield-Moore et al., 2005). In addition, studies showing comparable RE-induced MPS responses between young and older individuals report that younger adults achieve greater, or similar improvements in muscle strength and mass compared to their older counterparts following prolonged resistance training (Brook et al., 2016; Mayhew et al., 2009). Overall, there seems to be evidence pointing towards an age-related insensitivity to RE–induced stimuli, apparently most pronounced at lower exercise load/volume. Yet, the muscle of older adults retains substantial ability to mount robust synthetic and hypertrophic responses when provided with appropriately dosed exercise stimuli.

### Effect of age per se on exercise-induced protein synthesis in the protein-fed state

Across studies utilizing combination of exercise with protein or amino acid supplementation, most of the current evidence supports that post-exercise MPS in the fed state is largely preserved with aging (7 of 9 studies). An early tracer study demonstrated altered temporal kinetics in older adults, showing a delayed but not absent rise in post-exercise MPS following EAA ingestion compared with young adults (Drummond et al., 2008). This delay may reflect slower amino acid delivery or uptake rather than an intrinsic inability of aged muscles to respond to anabolic stimuli. Nevertheless, these are not uniform findings, supporting the notion of a state of context-dependent anabolic resistance. Of the 9 RE studies, the two that demonstrated a superior MPS response in young adults involved either moderate exercise volume (6 sets of 10 repetitions at 40–75% 1RM) combined with high protein intake (66 g protein and 12.3 g leucine), or high RE volume (8 sets of 10 repetitions at 70% 1RM) combined with moderate protein intake (20 g EAA). Both studies employed similar MPS measurement methodologies (phenylalanine tracer, mixed, intracellular/plasma) and had comparable sample sizes (6–8 participants). In contrast, the remaining studies utilized moderate to high RE volumes and moderate to high protein intakes (14.2–90 g), alongside similar MPS methodologies and sample sizes (**Supplementary Table S4**). No consistent patterns emerged across studies, aside from the observation that studies demonstrating a superior MPS response in young adults tended to be positioned at the high end of the exercise volume × protein intake spectrum. Our overall findings align with a previous systematic review [38], in which only two study arms demonstrated anabolic resistance in the fed post-exercise state, while eight reported neutral results. Collectively, this evidence indicates that post-exercise MPS is largely maintained with advancing age when RE is adequately dosed and given that protein consumption is of sufficient quantity, quality, and timing. Thus, the convergence of post-exercise, fed-state MPS responses between young and older adults may reflect a permissive or sensitizing effect of exercise in aging muscle, rather than a superior anabolic responsiveness. However, currently available data are insufficient to firmly determine whether true normalization of anabolic sensitivity occurs.

RE seems to provide a potent anabolic signal in both young and older adults (Fry et al., 2011; Kumar et al., 2009; Yang et al., 2012a). Noteworthy, RE performed in combination with protein feeding seem to prime the muscle to utilize amino acids more efficiently, suggesting that mechanical loading sensitizes aged muscles to the subsequent nutrient stimulus (Hermans et al., 2023; Pennings et al., 2011; Symons et al., 2011). Protein dose and quality further modulate the magnitude of the MPS response to combined feeding and exercise. Leucine enriched or high-quality protein in adequate doses (>20-30g) tend to restore MPS in older adults to levels comparable with young individuals (Atherton et al., 2017; Hermans et al., 2023; Horwath et al., 2025). Conversely, when protein doses are marginal or leucine content low, older muscles may fail to reach the threshold for additive MPS stimulation (Yang et al., 2012a, Yang et al., 2012b). In line with this, Atherton et al. (2017) observed that leucine supplementation enhanced p70S6K1 phosphorylation in older but not young, suggesting that older muscle retains sensitivity to leucine-mediated signaling but may require a stronger intracellular leucine signal to fully activate downstream translation pathways. Furthermore, a study reported an additive effect on MPS of co-ingestion of 1.5g leucine to 15g of protein supplementation after performing a single bout of RE in elderly males (Holwerda et al., 2019). Interestingly, there appears to be a clear dose–response relationship between protein intake (10–40 g) and MPS in elderly adults when high quality protein supplementation is combined with RE (Yang et al., 2012a, Yang et al., 2012b). This stands in contrast to protein supplementation alone, where MPS seems to plateau at a relatively low protein dose of around 10 g (Cuthbertson et al., 2005). Similarly, whole meal feeding studies further support that mixed meals containing sufficient protein can stimulate acute MPS similarly in older and younger adults (Phillips et al., 2017; Symons et al., 2011).

### Effect of age on muscle protein breakdown

Most studies, including measures of MPB, indicate that it is largely unaffected by age across post-prandial, post-exercise and combined conditions. These findings largely support the notion that age-related differences in muscle protein turnover are primarily driven by reductions in MPS rather than elevations in MPB in healthy adults, as suggested previously (Brook et al., 2022; Morton et al., 2018a; Phillips et al., 2009; Wilkinson et al., 2018). Consequently, strategies to mitigate primary sarcopenia should focus on enhancing the anabolic response to nutritional and mechanical stimuli rather than suppressing MPB.

## Conclusions and limitations

Overall, while basal and nutrient-stimulated MPS decline modestly with age, responsiveness to exercise alone or combined with protein intake appears mixed, with evidence generally suggesting a maintained anabolic potential when sufficient stimuli are provided. Consequently, the collectively observed age-related attenuation in MPS, when extrapolated from short-term measurements (<24 hours) to longer timescales - days, months, or years - may translate into substantial muscle loss over time, potentially serving as a key contributor to age-related muscle decline, i.e., sarcopenia. However, emerging data indicate that maximizing MPS alone is insufficient to prevent age-related losses in muscle function. As such, MPS should be viewed as a necessary but not exclusive determinant of muscle health, operating alongside other important proteostatic factors (Fuqua et al., 2023; Ubaida-Mohien et al., 2019). Noteworthy, age-related anabolic resistance seems to appear mostly when the combined stimuli is weak. As such, older individuals appear to require a stronger mechanical and/or nutritional stimuli to achieve the same MPS response as younger individuals.

Overall, discrepancies across studies may be due to methodological differences (e.g., tracer type, infusion duration, tissue analyzed), variability in physical activity and nutritional status, or inter-individual heterogeneity in aging trajectories. There was considerable variability in the duration of amino acid tracer infusion across the included studies. This variation may affect comparability, as MPS responses are time-dependent, and could contribute to heterogeneity in reported outcomes.

Furthermore, the overall quality of evidence varied across studies, as indicated by our ROBINS-I risk of bias assessment. Common concerns related to incomplete characterization of participants, particularly regarding habitual diet, physical activity level and sex distribution between age groups, as well as limited control of pre-study physical activity and nutritional status. Importantly, inconsistent reporting of these factors also limited the ability to formally evaluate them as potential moderators of age-related differences in MPS responses, thereby constraining interpretation of between-study heterogeneity. These issues may have influenced the observed variability across studies and should be considered when interpreting the strength and consistency of the age-related differences in MPS outcomes and when designing future trials.

## Knowledge gaps

While extensive research on MPS in aging, some important answers remain unanswered that limit the understanding of how age per se affects protein turnover. Firstly, the majority of studies have focused on healthy, lean older adults, creating uncertainty regarding the applicability of findings to more heterogeneous populations, including frail, sarcopenic, obese, or chronically ill individuals. These populations may exhibit more pronounced anabolic resistance, but seem underrepresented in mechanistic studies, leaving a gap in understanding the full spectrum of age-related MPS changes. Moreover, the role of habitual diet and habitual physical activity patterns has been inconsistently reported or controlled, confounding and complicating interpretation of age-related changes in both basal and post-prandial MPS.

As for design and methodological approach, differences and inconsistencies exist that contribute to challenging interpretation across studies. In accordance, RE stands out as the most potent anabolic stimulus, yet few studies compare the acute responses to differentiated exercise types. The sensitivity of an anabolic response to exercise stimulation may also depend on the level of accustomization (none to high) to the applied stimulus, as it may accordingly provoke an un-representative response (overly pronounced or attenuated) compared to a more average expectancy. Furthermore, previous studies employ diverse tracer techniques (e.g., isotope marked amino acids versus D_2_O), sampling windows, and differ in whether mixed-muscle or subfraction (myofibrillar, mitochondrial, collagen) protein synthesis are assessed. This makes it difficult to judge if different sub-fractions exhibit similar sensitivity or temporal response with increased age. Also, acute measurements over short post-prandial or post-exercise windows may fail to capture differential temporal MPS responses between age groups, potentially over-/underestimating synthetic capacity. In this regard, integrated tracer methods that assess cumulative synthesis over days or weeks are still scarce. A similar scarcity exists in measurements of protein degradation rates that run parallel with MPS, which limits insight into net protein balance and the regulation of proteolytic pathways with aging. This together with the majority of available data derived from short-term or acute interventions, leaves uncertainty regarding the translation of acute MPS responses to long-term changes in muscle mass, strength, and functional capacity.

As for a mechanistic understanding of potential anabolic resistance, reduced sensitivity likely reflects a combination of intrinsic and extrinsic mechanistic explanatory factors. At the signaling level, aging may impair canonical anabolic pathways such as mTORC1 and downstream effectors (e.g. p70S6K1, rpS6, 4E-BP1) (Fry et al., 2011; Guillet et al., 2004), although some evidence point towards retained responsiveness to acute anabolic stimuli (Brook et al., 2016; Drummond et al., 2008; Horwath et al., 2025). Specifically, phosphorylation of p70S6K1 was augmented only at higher volumes or intensities with aging, suggesting that aged muscle retains the molecular machinery for protein synthesis but requires a stronger contractile signal for full activation (Kumar et al., 2012, Kumar et al., 2009). While deficits in mTORC1/S6K1 signaling have been reported, complementary pathways including MAPK/ERK, AMPK–PGC-1α, eIF2α/GCN2, ribosome biogenesis, satellite cell signaling, and hormonal regulators that collectively integrate mechanical, metabolic, and nutrient cues to regulate muscle growth and adaptation (Schiaffino et al., 2013; Vainshtein and Sandri, 2020) remain poorly characterized.

Other influential factors may be age-related changes in ribosomal biogenesis and the RNA/DNA ratio that may limit translational capacity, reducing the efficiency of protein synthesis in aged muscle (Brook et al., 2016; Cuthbertson et al., 2005; Stec et al., 2015), while impaired transcriptional regulation may result in age-related reductions in the expression of genes involved in growth and repair (Deane et al., 2021; Dennis et al., 2008). Furthermore, age-related changes in mitochondrial function and microvascular perfusion, which influence amino acid delivery and utilization, are not commonly integrated into experimental designs. As for extrinsic factors, age-related chronic low-grade inflammation (“inflammaging”) may interfere with the regulation of MPS through crosstalk between pro-inflammatory and anabolic signaling pathways (Beyer et al., 2012; Ferrucci and Fabbri, 2018).

Hormonal milieu may also influence anabolic potential. Acute exercise-induced hormonal responses do not predict training-induced protein accretion or acute MPS in either young and elderly males (Brook et al., 2016; Morton et al., 2018a; West et al., 2009). However, circulating sex hormones decline with age (Tepper et al., 2012; Travison et al., 2007), which may contribute to reduced anabolic potential. Testosterone’s stimulatory effect on MPS (Brodsky et al., 1996; Smith et al., 2014) and muscle accretion appears well-documented, with testosterone restoration in hypogonadal older men increasing muscle mass, both with and without concurrent resistance-training interventions (Emmelot-Vonk et al., 2008; Kvorning et al., 2013; Skinner et al., 2018). In contrast, the role of estradiol remains less clear, as basal MPS has been reported to be higher in post- vs. pre-menopausal women with no consistent effect of estradiol administration or menstrual cycle phase (Apicella et al., 2026; Hansen et al., 2012; Smith et al., 2014). However, prolonged hormone replacement therapy combined with RE appear to directly enchance muscle adaptations compared to placebo (Dam et al., 2020). Finally, age-related fast-twitch fibre atrophy and loss, shifting towards a relative increase in type I fibre proportion (number/area) (Sonjak et al., 2019; Tøien et al., 2023), likely influences interpretations, as type II fibers are intrinsically more responsive to anabolic stimuli (Edman et al., 2019; Koopman et al., 2006b; Roberts et al., 2020), while age-related neuromuscular deficits may limit type II fibre exercise-induced recruitment (Hunter et al., 2016).

## Considerations for future directions

Addressing the current knowledge gaps requires studies that integrate methodological rigor with translational relevance. Future research should include diverse populations, encompassing frail, sarcopenic, obese, or chronically ill older adults, alongside healthy controls. Stratification by activity patterns and habitual diet will help identify population-specific patterns of anabolic resistance. Acute studies could benefit from including accustomization regimes prior to the actual exercise experiments and longitudinal studies could track changes in MPS, muscle mass, strength, and functional outcomes over months or even years to better determine the trajectory of age-related alterations and their clinical implications.

Methodologically, studies could employ tracer techniques that are capable of providing information on subfraction protein synthesis and seek to combine with measures of protein degradation to estimate net balance. Additionally, incorporating assessments of microvascular perfusion, mitochondrial function, and amino acid delivery alongside MPS measurements can help clarify the underlying physiological mechanisms. Standardization of protein feeding protocols, including dose, leucine content, and timing relative to exercise, is essential for interpreting the effects of nutrition on MPS and mitigating variability across studies. Finally, mechanistic studies should expand beyond canonical mTORC1/S6K1 signaling, investigating alternative anabolic pathways and the regulation of autophagy and proteolysis.

## Data Availability

The original contributions presented in the study are included in the article/**Supplementary Material**. Further inquiries can be directed to the corresponding author.
