# Supplementary material for: Age-related anabolic resistance and post-absorptive muscle protein synthesis: integrative evidence from a systematic review and meta-analysis
Source: Front Physiol. 2026 Jun 5;17:1740284. doi: 10.3389/fphys.2026.1740284 (PMC13278896; doi:10.3389/fphys.2026.1740284)
Supplement: Supplementary file 2 [file Image2.pdf]

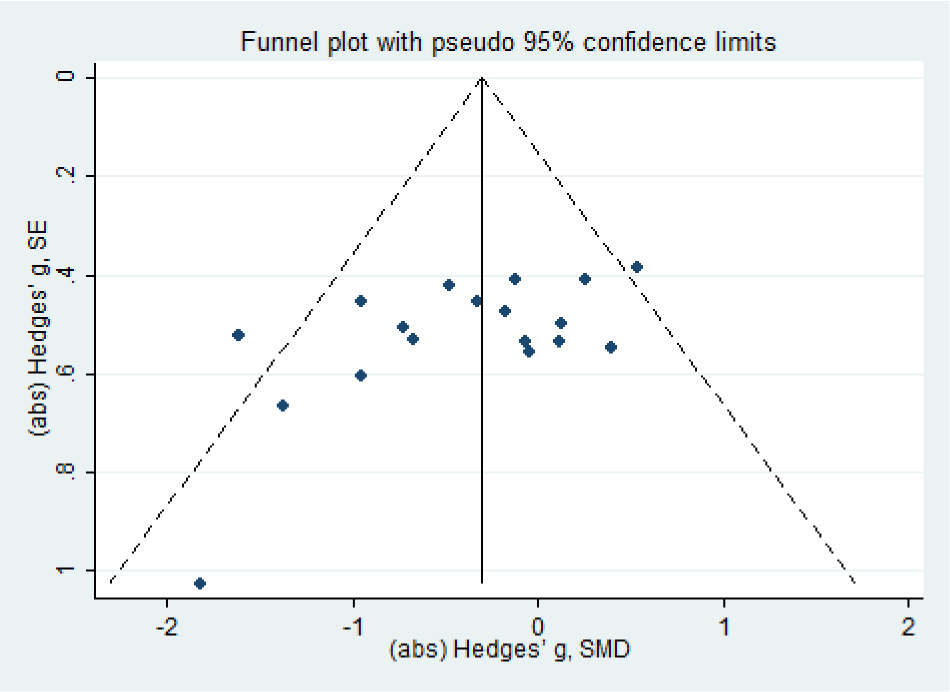


**Figure S2.** Funnel plot of studies assessing post-prandial MPS. Effect sizes are presented as standardized mean differences (Hedges' g) on the x-axis, and standard errors (Hedges' g) on the y-axis. The vertical solid line represents the pooled overall effect estimate, while the dashed lines indicate pseudo 95% confidence limits. Each square represents individual studies. Visual inspection of the plot suggests a relatively symmetrical distribution of studies around the pooled effect size, with no strong indication of publication bias or small-study effects.
