# Supplementary material for: Age-related anabolic resistance and post-absorptive muscle protein synthesis: integrative evidence from a systematic review and meta-analysis
Source: Front Physiol. 2026 Jun 5;17:1740284. doi: 10.3389/fphys.2026.1740284 (PMC13278896; doi:10.3389/fphys.2026.1740284)
Supplement: Supplementary file 3 [file Table1.pdf]

Reference	Study design	Sample size, n (n, females)	Age (yrs)	Habitual condition	Method for MPS	Protocol for MPS assessment	Postabsorptive MPS	Group difference	Notes / Additional outcomes
Atherton et al. (2017)	NR-PGD, AGR	18 (0) / 18 (0)	70 ± 5 / 24 ± 6	Healthy, physical activity level NA	[1,2-$^{13}C_2$]-leucine, myo, plasma	Infusion bolus (0h; 0.7 mg/kg) and rate (1.0 mg/kg/h)<br>Biopsy (0h and 2.5h)<br>MPS duration 2.5h	Protein + leucine/alanine 0.039 ± 0.004 / 0.040 ± 0.002	Protein + leucine / alanine Y = O (-2%)	Similar postabsorptive MPS values in Y and O<br>Similar phosphorylation of p70S6K in Y and O
Babraj et al. (2005)	NR-PGD, AGR	8 (0) / 8 (0)	70 ± 2 / 28 ± 2	Healthy, physical activity level NA	L-[1-^{13}C]-leucine, myo, plasma	Infusion bolus (0h; 0.8 mg/kg) and rate (1.0 mg/kg/min)<br>Biopsy (0h and 4h)<br>MPS duration 4h	0.029 ± 0.005 / 0.032 ± 0.004	Y = O (10%)	Similar postabsorptive MPS values in Y and O<br>Muscle collagen FSR was greater in O vs. Y
Balagopal et al. (1997)	NR-PGD	8 (4) / 8 (4)	77 ± 2 / 23 ± 1	Healthy, low physical activity level	L-[1-^{13}C]-leucine, mixed, plasma	Infusion bolus (0h; 7 µmol/kg) and rate (7.5 µmol/kg/min)<br>Biopsy (5h and 10h)<br>MPS duration 5h	0.036 ± 0.002 / 0.047 ± 0.003	Y > O (31%)*	Greater postabsorptive MPS in Y vs. O
Chevalier et al. (2011)	NR-PGD	8 (8) / 8 (8)	73 ± 3 / 24 ± 1	Healthy, low physical activity level	L-[ring-$^{2}H_5$]-phenyl-alanine, mixed, IC	Infusion bolus (0h; 0.89 mg/kg) and rate (0.026 mg/kg/min)<br>Biopsy (5h and 7h)<br>MPS duration 2h	0.041 ± 0.003 / 0.038 ± 0.003	Y = O (-7%)	Similar postabsorptive MPS values in Y and O<br>Similar phosphorylation of Akt/mTORC1 signalling in Y and O
Cuthbertson et al. (2005)	NR-PGD	24 (0) / 20 (0)	70 ± 1 / 28 ± 1	Healthy, physical activity level NA	[1-^{13}C]-KIC, myo, IC	Infusion bolus (0h; 8.8 µmol/kg) and rate (13.2 µmol/kg/min)<br>Biopsy (0.5h and 3h)<br>MPS duration 2.5h	0.032 ± 0.005 / 0.032 ± 0.004	Y = O (0%)	Similar postabsorptive MPS values in Y and O<br>Similar phosphorylation of, mTOR and p70S6K in Y and O
Dillon et al. (2011)	NR-PGD	7 (4) / 7 (5)	67 ± 2 / 30 ± 2	Healthy, physical activity level NA/no regular exercise	L-[ring-$^{13}C_6$]-phenyl-alanine, mixed, IC	Infusion bolus (0h; 2.0 µmol/kg) and rate (0.08 µmol/kg/min)<br>Biopsy (2h and 4h)<br>MPS duration 2h	0.070 ± 0.004 / 0.067 ± 0.009	Y = O (-4%)	Similar postabsorptive MPS values in Y and O<br>Similar phosphorylation of mTOR and AMPK in Y and O
Drummond et al. (2008)	NR-PGD	6 (0) / 7 (0)	70 2 / 30 ± 2	Healthy, physical activity level NA/no regular exercise	L-[ring-$^{2}H_5$]-phenyl-alanine, mixed, IC	Infusion bolus (0h; 2.0 µmol/kg) and rate (0.05 µmol/kg/min)<br>Biopsy (2h and 3h)	0.046 ± 0.006 / 0.044 ± 0.007	Y = O (-4%)	Similar postabsorptive MPS values in Y and O<br>Similar phosphorylation of Akt, mTOR, p70S6K and

						MPS duration 1h			4E-BPI1 in Y and O
Durham et al. (2010)	NR-PGD	8 (0) / 9 (0)	67 ± 2 / 30 ± 2	Healthy, physical activity level NA/no regular exercise	L-[ring-$^{2}H_{5}$]-phenyl-alanine, mixed, IC	Infusion bolus (0h; 2.0 µmol/kg) and rate (0.05 µmol/kg/min)<br>Biopsy (1h45min, 3h45min)<br>MPS duration 2h	0.070 ± 0.006 / 0.061 ± 0.005	Y = O (-13%)	Similar postabsorptive MPS values in Y and O<br>Similar phosphorylation of Akt, mTOR, p70S6K and 4E-BPI1 in Y and O
Fry et al. (2011)	NR-PGD	16 (8) / 16 (8)	70 ± 2 / 27 ± 2	Healthy, recreationally active/no regular exercise	L-[ring-$^{13}C_{6}$]-phenyl-alanine, mixed, IC	Infusion bolus (0h; 2.0 µmol/kg) and rate (0.05 µmol/kg/min)<br>Biopsy (2h and 4h)<br>MPS duration 2h	0.054 ± 0.003 / 0.052 ± 0.004	Y = O (-4%)	Similar postabsorptive MPS values in Y and O<br>Similar phosphorylation of Akt, mTOR, p70S6K, 4E-BPI1, rpS6 and ERK1/2 in Y and O
Gorissen et al. (2014)	NR-PGD, AGR	13 (0) / 12 (0)	76 ± 1 / 20 ± 1	Healthy, physical activity level NA/no regular exercise	L-[ring-$^{2}H_{5}$]-phenyl-alanine, mixed, plasma	Infusion bolus (0h; 2.0 µmol/kg) and rate (0.05 µmol/kg/min)<br>Biopsy (1.5h and 3.5h)<br>MPS duration 2h	0.029 ± 0.002 / 0.029 ± 0.003	Y = O (-3%)	Similar postabsorptive MPS values in Y and O
Groen et al. (2016)	NR-PGD	24 (0) / 24 (0)	68 ± 1 / 22 ± 1	Healthy, physical activity level NA	L-[ring-$^{2}H_{5}$]-phenyl-alanine, mixed, plasma	Infusion bolus (0h; 2.0 µmol/kg) and rate (0.05 µmol/kg/min)<br>Biopsy (3h and 5h)<br>MPS duration 2h	0.024 ± 0.002 / 0.030 ± 0.002	Y > O (28%)*	Greater postabsorptive MPS in Y vs. O
Guillet et al. (2004)	NR-PGD	8 (NA) / 6 (NA)	72 ± 2 / 25 ± 1	Healthy, physical activity level NA	L-[1-^{13}C]-leucine, mixed, IC	Infusion bolus (0h; 8.4 µmol/kg FFM) and rate (0.14 µmol/kg FFM/min)<br>Biopsy (4h and 8h)<br>MPS duration 4h	0.061 ± 0.004 / 0.082 ± 0.010	Y > O (34%) *	Greater postabsorptive MPS in Y vs. O
Hasten et al. (2000)	NR-PGD	7 (3) / 7 (4)	80 ± 1 / 27 ± 1	Healthy, physical activity level NA	[1-^{13}C]-leucine, mixed, plasma	Infusion bolus (0h; 7.58 µmol/kg) and rate (7.58 µmol/kg/h)<br>Biopsy (1.5h and 14h)	0.037 ± 0.003 / 0.048 ± 0.003	Y > O (30%)	Similar postabsorptive MPS values in Y and O

						MPS duration 12.5h			
Henderson et al. (2009)	NR-PGD, AGR	144 (57) /<br>62 (32)	Male:<br>69 ± 1<br>23 ± 1<br><br>Female:<br>70 ± 1 /<br>21 ± 0	Healthy, physical activity level NA/no regular exercise	[^{15}N]-phenyl-alanine, mixed, plasma	Infusion bolus (0h; 0.75 mg/kg) and rate (0.75 mg/kg/h)<br>Biopsy (3h and 8h)<br>MPS duration 5h	Male<br>0.027 ± 0.001 /<br>0.030 ± 0.001<br>Female<br>0.031 ± 0.001 /<br>0.035 ± 0.001	Male<br>Y > O (10%)*<br>Female<br>Y > O (12%)*	Greater postabsorptive MPS in Y vs. O in both male and female.<br>MPS was generally greater in female vs. male
Hermans et al. (2023)	NR-PGD	15 (0) /<br>14 (0)	73 ± 1 /<br>25 ± 1	Healthy, physical activity level NA/no to low regular exercise and no RT	L-[ring-$^{13}C_6$]-phenyl-alanine, mixed, plasma	Infusion bolus (0h; 2.25 µmol/kg) and rate (0.05 µmol/kg/min)<br>Biopsy (1h and 3.5h)<br>MPS duration 2.5h	0.036 ± 0.003 /<br>0.030 ± 0.001	Y = O (-17%)	Similar postabsorptive MPS values in Y and O
Horwath et al. (2025)	NR-PGD	10 (0) /<br>10 (0)	70 ± 1 /<br>22 ± 1	Healthy, recreationally active/no RT	L-[ring-$^{13}C_6$]-phenyl-alanine, myo, IC	Infusion bolus (0h; 2.0 µmol/kg) and rate (0.05 µmol/kg/min)<br>Biopsy (2.5h)<br>MPS duration 2.5h	IC<br>0.050 ± 0.006 /<br>0.058 ± 0.007	IC<br>Y = O (16%)	Similar postabsorptive MPS values in Y and O in both precursor pools<br>Greater phosphorylation of rpS6$^{Ser240/244}$ in O vs Y
Katsanos et al. (2006)	NR-PGD, AGR	20 (8) /<br>16 (8)	68 ± 2 /<br>30 ± 2	Healthy, physical activity level NA/no regular exercise	L-[ring-$^{2}H_5$]-phenyl-alanine, mixed, plasma	Infusion bolus (0h; 2.0 µmol/kg) and rate (0.05 µmol/kg/min)<br>Biopsy (1h and 4h)<br>MPS duration (4h)	0.041 ± 0.04 /<br>0.042 ± 0.03	Y = O (4)	Similar postabsorptive MPS values in Y and O in both conditions
Kumar et al. (2009)	NR-PGD, AGR	15 (0) /<br>15 (0)	70 ± 5 /<br>24 ± 6	Healthy, recreationally active	[1,2-$^{13}C_2$]-leucine, myo, plasma	Infusion bolus (0h; 0.7 mg/kg) and rate (1 mg/kg/h)<br>Biopsy (0h and 2.5h)<br>MPS duration (2.5h)	0.043 ± 0.003 /<br>0.040 ± 0.006	Y = O (-7%)	Similar postabsorptive MPS values in Y and O<br>Similar post/absorptive phosphorylation of P70S6K and 4E-BP1 in Y and O
Kumar et al. (2012)	NR-PGD, AGR, CO	24 (0) /<br>24 (0)	70 ± 5 /<br>24 ± 6	Healthy, recreationally active	[1,2-$^{13}C_2$]-leucine, myo, plasma	Infusion bolus (0h; 0.7 mg/kg) and rate (1 mg/kg/h)<br>Biopsy (0h and 2.5h)	0.039 ± 0.003 /<br>0.042 ± 0.002	Y = O (8%)	Similar postabsorptive MPS values in Y and O in all conditions<br>Similar postabsorptive phosphorylation of

						MPS duration (2.5h)			P70S6K and 4E-BP1 in Y and O
Lalia et al. (2017)	NR-PGD	12 (7) / 12 (6)	76 ± 1 / 27 ± 1	Healthy, physical activity level NA/no to a low volume of regular exercise	L-[ring-$^{13}C_6$]-phenyl-alanine, mixed, IC	Infusion bolus (0h; 1.5 mg/kg FMM) and rate (1.5 mg/kg FFM/h)<br>Biopsy (3hh and 6hh)<br>MPS duration (3hh)	0.072 ± 0.007 / 0.067 ± 0.006	Y = O (-7%)	Similar postabsorptive MPS values in Y and O in both muscle fractions
Mayhew et al. (2009)	NR-PGD	6 (NA) / 8 (NA)	64 ± 1 / 27 ± 1	Healthy, physical activity level NA/no RT	L-[ring-$^{2}H_5$]-phenyl-alanine, mixed, IC	Infusion bolus (0h; 2.0 µmol/kg) and rate (0.05 µmol/kg/min)<br>Biopsy (0h and 24h)<br>MPS duration (24h)	0.052 ± 0.010 / 0.055 ± 0.006	Y = O (6%)	Similar postabsorptive MPS values in Y and O<br>Similar postabsorptive phosphorylation of P70S6K, rpS6 and 4E-BP1 in Y and O
Mitchell et al. (2017)	NR-PGD	8 (0) / 8 (0)	70 ± 1 / 20 ± 1	Healthy, physical activity level NA	L-[ring-$^{13}C_6$]-phenyl-alanine, myo, IC	Infusion bolus (0h; 0.3 mg/kg) and rate (0.6 mg/kg/h)<br>Biopsy (1h and 2h)<br>MPS duration (1h)	0.051 ± 0.004 / 0.054 ± 0.005	Y = O (6%)	Similar postabsorptive MPS values in Y and O
Paddon-Jones et al. (2004)	NR-PGD	7 (4) / 6 (4)	67 ± 1 / 34 ± 2	Healthy, recreationally active/no regular exercise	L-[ring-$^{2}H_5$]-phenyl-alanine, mixed, IC	Infusion bolus (0h; 2.0 µmol/kg) and rate (0.05 µmol/kg/min)<br>Biopsy (2h and 5h)<br>MPS duration 3h	0.056 ± 0.004 / 0.064 ± 0.007	Y = O (14%)	Similar postabsorptive MPS values in Y and O
Phillips et al. (2017)	NR-PGD	17 (50/50) / 11 (50/50)	70 ± 3 / 24 ± 1	Healthy, physical activity level NA/no regular exercise	[1,2-$^{13}C_2$]-leucine, myo, plasma	Infusion bolus (0h; 0.66 mg/kg) and rate (1.0 mg/kg/h)<br>Biopsy (0h and 2.5h)<br>MPS duration (2.5h)	0.042 ± 0.003 / 0.044 ± 0.004	Y = O (5%)	Similar postabsorptive MPS values in Y and O
Rasmussen et al. (2006)	NR-PGD	6 (1) / 7 (4)	68 ± 1 / 25 ± 2	Healthy, recreationally active/no regular exercise	L-[ring-$^{2}H_5$]-phenyl-alanine, mixed, IC	Infusion bolus (0h; 2.0 µmol/kg) and rate (0.05 µmol/kg/min)<br>Biopsy (2h and 5h)<br>MPS duration 3h	0.065 ± 0.003 / 0.070 ± 0.003	Y = O (8%)	Similar postabsorptive MPS values in Y and O

Rooyackers et al. (1996)	NR-PGD	16 (9) / 12 (6)	73 ± 2 / 24 ± 1	Healthy, physical activity level NA/no to a low volume of regular exercise	L-[1-^{13}C]-leucine, mixed, plasma	Infusion bolus (0h; NA) and rate (7.5 µmol/kg/h)<br>Biopsy (5h and 10h)<br>MPS duration 5h	0.038 ± 0.003 / 0.043 ± 0.002	Y > O (13%)*	Greater postabsorptive MPS in Y vs. O
Sheffield-Moore et al. (2005)	NR-PGD	6 (0) / 6 (0)	67 ± 2 / 27 ± 3	Healthy, physical activity level NA/no regular exercise	L-[ring-$^{2}H_{5}$]-phenyl-alanine, mixed, IC	Infusion bolus (0h; 2.0 µmol/kg) and rate (0.05 µmol/kg/min)<br>Biopsy (3h)<br>MPS duration 3h	0.076 ± 0.004 / 0.072 ± 0.003	Y = O (-5%)	Similar postabsorptive MPS values in Y and O
Smeuninx et al. (2017)	NR-PGD	17 (9) / 18 (8)	70 ± 1 / 25 ± 1	Healthy, recreationally active/no regular exercise	L-[ring-$^{13}C_{6}$]-phenyl-alanine, myo, plasma	Infusion bolus (0h; 2.0 µmol/kg) and rate (0.05 µmol/kg/min)<br>Biopsy (2.5h)<br>MPS duration 2.5h	0.031 ± 0.002 / 0.027 ± 0.002	Y = O (-15%)	Similar postabsorptive MPS values in Y and O
Symons et al. (2009)	NR-PGD, AGR	17 (7) / 17 (9)	68 ± 1 / 35 ± 1	Healthy, recreationally active/no regular exercise	L-[ring-$^{13}C_{6}$]-phenyl-alanine, mixed, IC	Infusion bolus (0h; 2.0 µmol/kg) and rate (0.08 µmol/kg/min)<br>Biopsy (3h and 6h)<br>MPS duration 3h	0.082 ± 0.004 / 0.081 ± 0.006	Y = O (-1%)	Similar postabsorptive MPS values in Y and O in both conditions
Toth et al. (2005)	NR-PGD	15 (5) / 7 (4)	72 ± 1 / 29 ± 2	Healthy, sedentary to recreationally active	[1,2-$^{13}C_{2}$]-leucine, mixed, plasma	Infusion bolus (0h; 4.8 µmol/kg) and rate (5.6 µmol/kg/min)<br>Biopsy (1.5h and 8h)<br>MPS duration 6.5h	0.037 ± 0.003 / 0.045 ± 0.005	Y > O (23%)*	Greater postabsorptive MPS in Y vs. O
Volpi et al. (1999)	NR-PGD	8 (2) / 7 (3)	71 ± 2 / 30 ± 2	Healthy, recreationally active/no regular exercise	L-[ring-$^{2}H_{5}$]-phenyl-alanine, mixed, IC	Infusion bolus (0h; 2.0 µmol/kg) and rate (0.05 µmol/kg/min)<br>Biopsy (2h and 5h)<br>MPS duration 3h	0.050 ± 0.009 / 0.044 ± 0.004	Y = O (-12%)	Similar postabsorptive MPS values in Y and O
Volpi et al. (2000)	NR-PGD	5 (1) / 5 (3)	72 ± 1 / 30 ± 3	Healthy, recreationally active/no regular	L-[ring-$^{2}H_{5}$]-phenyl-alanine, mixed, IC	Infusion bolus (0h; 2.0 µmol/kg) and rate (0.05 µmol/kg/min)	0.06 ± 0.011 / 0.06 ± 0.009	Y = O (0%)	Similar postabsorptive MPS values in Y and O

				exercise		Biopsy (2h and 5h)<br>MPS duration 3h			
Volpi et al. (2001)	NR-PGD	22 (0) /<br>26 (0)	80 ± 3 /<br>28 ± 2	Healthy, recreationally active/no regular exercise	L-[ring-$^{2}H_{5}$]-phenyl-alanine, mixed, plasma	Infusion bolus (0h; 2.0 µmol/kg) and rate (0.05 µmol/kg/min)<br>Biopsy (2h and 5h)<br>MPS duration 3h	0.06 ± 0.005 /<br>0.058 ± 0.005	Y = O (-4%)	Similar postabsorptive MPS values in Y and O
Walrand et al. (2008)	NR-PGD	18 (9) /<br>20 (10)	70 ± 1.8 /<br>24 ± 1	Healthy, physical activity level NA/no to a small volume of regular exercise	L-[1-^{13}C]-leucine, mixed, plasma	Infusion bolus (0h; NA) and rate (7.5 µmol/kg FFM/min)<br>Biopsy (3h and 8h)<br>MPS duration 5h	0.032 ± 0.003 /<br>0.040 ± 0.004	Y = O (25%)	Similar postabsorptive MPS values in Y and O in both conditions<br>Data obtained after 10 days of either usual protein intake or high protein intake
Welle et al. (1993)	NR-PGD	8 (0) /<br>8 (0)	68 ± 2 /<br>26 ± 1	Healthy, moderate physical active/no RT	[1-^{13}C]-leucine, myo, plasma	Infusion bolus (0h; NA) and rate (NA)<br>Biopsy (0h and 8h) - for n=3 in each group at 2h and 8h<br>MPS duration 8h (and 6h)	0.040 ± 0.009 /<br>0.054 ± 0.010	Y > O (37%)*	Greater postabsorptive MPS in Y vs. O
Welle et al. (1995)	NR-PGD	9 (4) /<br>9 (4)	67 ± 1/<br>27 ± 1	Healthy, low to high physical active/no RT	[1-^{13}C]-leucine, myo, plasma	Infusion bolus (0h; NA) and rate (NA)<br>Biopsy (2h and 8h)<br>MPS duration 6h	0.041 ± 0.005 /<br>0.061 ± 0.004	Y > O (49%)*	Greater postabsorptive MPS in Y vs. O
Yarasheki et al. (1993)	NR-PGD	6 (2) /<br>6 (4)	male:<br>63 ± 1 /<br>24 ± 0<br><br>female:<br>67 ± 7 /<br>24 ± 1	Healthy, physical activity level NA	[1-^{13}C]-leucine & [1,2-$^{13}C_{2}$]-leucine, mixed, plasma	Infusion bolus (0h; 7.58 µmol/kg) and rate (7.58 µmol/kg/h)<br>Biopsy (~1.5-2h and 6h)<br>MPS duration ~4-4.5h	0.030 ± 0.004 /<br>0.049 ± 0.005	Y > O (66%)*	Greater postabsorptive MPS in Y vs. O

Table S1 - Schematic overview of studies involving post-absorptive muscle protein synthesis

Study design: Non-randomized parallel group design (NR-PGD), age-group randomization (AGR), cross-over (CO). Method for MPS: Type of tracer / type of MPS subfraction / type of precursor pool. Protocol for MPS assessment: Infusion details / muscle biopsy timing / MPS duration (timing relative to infusion initiation). Postabsorptive MPS: Absolute scores, unit: %-hrs. Group difference: Direction (%-difference relative to old postabsorptive scores), * denotes $P < 0.05$ as reported by the given study. All data are means ± SE and order-listed as old / young. FFM = fat free mass, IC = intracellular, KIC = ketoisocaproate, MPS = muscle protein synthesis, Myo = myofibrillar, O = old, RT = resistance training, Y = young.
