# Supplementary material for: Age-related anabolic resistance and post-absorptive muscle protein synthesis: integrative evidence from a systematic review and meta-analysis
Source: Front Physiol. 2026 Jun 5;17:1740284. doi: 10.3389/fphys.2026.1740284 (PMC13278896; doi:10.3389/fphys.2026.1740284)
Supplement: Supplementary file 4 [file Table2.pdf]

Reference	Study design	Sample size, n (n, females)	Age (yrs)	Habitual condition	Condition (fasted/fed)	Nutritional protocol (type, dose)	Method for MPS	Protocol for MPS assessment	MPS response (absolute)	Group difference (A)	MPS response (Δ)	Group difference (B)	Notes / Additional outcomes
Babraj et al. (2005)	NR-PGD, AGR	4 (0) / 4 (0)	70 ± 2 / 28 ± 2	Healthy, physical activity level NA	Fasted	20g EAA, orally	[1-^{13}C]-KIC, myo, plasma	Infusion bolus (0h; 1.1 mg/kg) and rate (1.7 mg/kg/min)<br>Protein supple. (0.5h)<br>Biopsy (0h and 3h)<br>MPS duration 3h	0.074 ± 0.009 / 0.105 ± 0.009	Y > O (42%)*	0.045 / 0.073	Y > O (62%)*	MPS increased from postabsorptive values in both Y and O but more so in Y
Chevalier et al. (2011)	NR-PGD	8 (8) / 8 (8)	73 ± 3 / 24 ± 1	Healthy, low physical activity level	Fasted	Infusion clamps (i.v.)<br>Hyperinsulinemic (0,4 mU/kg LBM/min)<br>Hyperglycemic (8 mmol/L)<br>Hyperaminoacidemic (700µmol/L BCAA)	L-[ring-$^{2}H_{5}$]-phenyl-alanine, mixed, IC	Infusion bolus (0h; 0.89 mg/kg) and rate (0.026 mg/kg/min)<br>Protein supple. (3h)<br>Biopsy (5h and 7h)<br>MPS duration 2h	0.078 ± 0.020 / 0.070 ± 0.008	Y = O (-10%)	0.037 / 0.032	Y = O (-14%)	MPS increased from post-absorptive values in both Y and O<br>Comparable insulin sensitivity of glucose metabolism between Y and O<br>Similar phosphorylation of p70S6K1 and rpS6
Cuthbertson et al. (2005)	NR-PGD	24 (0) / 20 (0)	70 ± 1 / 28 ± 1	Healthy, low physical activity level	Fasted	2.5g EAA<br>5g EAA<br>10g EAA<br>20g EAA<br>40g EAA (only O), orally<br>Hyperinsulinemic clamp: (360 m IU·m^{-2} body surface area ·h^{-1})	[1-^{13}C]-KIC, myo, IC	Infusion bolus (0h; 8.8 µmol//kg) and rate (13.2 µmol//kg/h)<br>Protein supple. (0.5h)<br>Biopsy (0.5h and 3h)<br>MPS duration 2.5h	2.5g<br>0.054 ± 0.004 / 0.057 ± 0.003<br>5g<br>0.062 ± 0.005 / 0.072 ± 0.008<br>10g<br>0.069 ± 0.004 / 0.104 ±	2.5g<br>Y = O (6%)<br>5g<br>Y = O (16%)<br>10g<br>Y > O (51%)*<br>20g<br>Y > O (42%)*	2.5g<br>0.022 / 0.025<br>5g<br>0.030 / 0.040<br>10g<br>0.037 / 0.072<br>20g<br>0.040 / 0.070<br>40g<br>0.034 / NA	2.5g<br>Y = O (14%)<br>5g<br>Y = O (33%)<br>10g<br>Y > O (95%)*<br>20g<br>Y > O (75%)*	MPS increased similarly from postabsorptive values in both Y and O at 2.5 and 5g EAA.<br>MPS was greater in Y vs. O at 10g and 20g EAA.<br>Phosphorylation of mTOR and p70S6K increased in both Y and O in response to 10g EAA, but to a lesser

						Ocreotide: (1.8 mg·kg^{-1}·h^{-1})			0.004<br>20g<br>0.072 ± 0.004 / 0.102 ± 0.004<br>40g<br>0.066 ± 0.002 / NA				extent in O
Dillon et al. (2011)	NR-PGD	7 (4) / 7 (5)	67 ± 2 / 30 ± 2	Healthy, physical activity level NA/no regular exercise	Fasted	AA infusion (i.v.)<br>EAA and NEAA (0.45 ml/kg (prime), 1.35 ml/kg/h (continuous, 3h))<br>Pharmacological vasodilation	L-[ring-$^{13}C_6$]-phenyl-alanine, mixed, IC	Infusion bolus (0h; 2.0 µmol//kg) and rate (0.08 µmol//kg/min)<br>Protein supple. (5h till 7h)<br>Biopsy (4h and 7h)<br>MPS duration 3h	0-3h<br>0.094 ± 0.009 / 0.089 ± 0.008	Y = O (-6%)	0.024 / 0.022	Y = O (-9%)	MPS increased from post-absorptive values in both Y and O<br>Similar increases in phosphorylation of mTOR and AMPK in Y and O
Gorissen et al. (2014)	NR-PGD, AGR	13 (0) / 12 (0)	76 ± 1 / 20 ± 1	Healthy, physical activity level NA/no regular exercise	Fasted	20g Casein (1.4g leucin), orally<br>60g carbohydrate, orally	L-[ring-$^{2}H_5$]-phenyl-alanine, mixed, plasma	Infusion bolus (0h; 2.0 µmol//kg) and rate (0.05 µmol//kg/min)<br>Protein supple. (3.5h)<br>Biopsy (3.5h, 5.5h and 8.5)<br>MPS duration 2h and 5h	Pro, 0-3h<br>0.028 ± 0.005 / 0.040 ± 0.006<br>Pro/CHO, 0-3h<br>0.024 ± 0.004 / 0.041 ± 0.004<br>Pro, 0-5h<br>0.033 ± 0.002 / 0.035 ± 0.003<br>Pro/CHO, 0-5h<br>0.035 ± 0.003 / 0.042 ± 0.004	Pro, 0-3h<br>Y = O (44%)<br>Pro/CHO, 0-3h<br>Y = O (71%)<br>Pro, 0-5h<br>Y = O (7%)<br>Pro/CHO, 0-5h<br>Y = O (22%)	Pro, 0-3h<br>-0.005 / 0.013<br>Pro/CHO, 0-3h<br>-0.002 / 0.011<br>Pro, 0-5h<br>-0.005 / 0.013<br>Pro/CHO, 0-5h<br>-0.002 / 0.011	Pro, 0-3h<br>Y = O (1498%)<br>Pro/CHO, 0-3h<br>Y = O (298%)<br>Pro, 0-5h<br>Y = O (81%)<br>Pro/CHO, 0-5h<br>Y = O (174%)	MPS increased from post-absorptive values only in Y in both interventions

Groen et al. (2016)	NR-PGD	24 (0) / 24 (0)	68 ± 1 / 22 ± 1	Healthy, physical activity level NA	Fasted	20g Casein, orally<br>Insulin (0.30 mU/min/100 mL leg volume)	L-[ring-$^{2}H_{5}$]-phenyl-alanine, mixed, plasma	Infusion bolus (0h; 2.0 µmol/kg) and rate (0.05 µmol/kg/min)<br>Protein supple. (3h)<br>Biopsy (3h, 5h and 8h)<br>MPS duration 2h and 5h	Pro 0-2h 0.038 ± 0.004 / 0.046 ± 0.004<br>Pro/insulin 0-2h 0.033 ± 0.003 / 0.046 ± 0.008<br>Pro 0-5h 0.037 ± 0.002 / 0.044 ± 0.004<br>Pro/insulin 0-5h 0.037 ± 0.002 / 0.050 ± 0.006	Pro, 0-2h Y > O (22%)*<br>Insulin, 0-2h Y > O (40%)*<br>Pro, 0-5h Y > O (19%)*<br>Pro/insulin, 0-5h Y > O (35%)*	Pro, 0-2h 0.015 / 0.017<br>Pro/insulin, 0-2h 0.009 / 0.015<br>Pro, 0-5h 0.014 / 0.015<br>Pro/insulin, 0-5h 0.013 / 0.019	Pro, 0-2h Y > O (15%)*<br>Pro/insulin, 0-2h Y > O (67%)*<br>Pro, 0-5h Y > O (7%)*<br>Pro/insulin, 0-5h Y > O (46%)*	MPS increased from postabsorptive values in Y and O with and without insulin, but more so in Y in both conditions<br>No effect of insulin administration (local insulin administration did not further increase postprandial MPS)
Guillet et al. (2004)	NR-PGD	8 (NA) / 6 (NA)	72 ± 2 / 25 ± 1	Healthy, physical activity level NA	Fasted	Hyperaminoacidemic clamp (i.v.) ~41g EAA<br>Hyperinsulinemic clamp (0.7 mUI/kg FFM/min)<br>Euglycemic clamp (20% glucose)	L-[1-^{13}C]-leucine, mixed, IC	Infusion bolus (0h; 8.4 µmol/kg FFM) and rate (0.14 µmol/kg FFM/min)<br>Protein supple. (4h-8h)<br>Biopsy (4h and 8h)<br>MPS duration 4h	0.084 ± 0.005 / 0.119 ± 0.006	Y > O (42%)*	0.023 / 0.037	Y > O (61%)*	MPS increased from postabsorptive values in both Y and O, but more so in Y<br>Similar phosphorylation of Akt, mTOR, and 4E-BP1 in Y and O<br>Phosphorylation of p70S6K increased only in Y
Hermans et al. (2023)	NR-PGD	15 (0) / 14 (0)	73 ± 1 / 25 ± 1	Healthy, physical activity level NA/no to a low volume of regular exercise and no RT	Fasted	30g of protein from Quark (dairy product), orally	L-[ring-$^{13}C_{6}$]-phenyl-alanine, mixed, plasma	Infusion bolus (0h; 2.25 µmol/kg) and rate (0.05 µmol/kg/min)<br>Protein supple. (3.5h)<br>Biopsy (3.5h and 7.5h)<br>MPS duration	0.062 ± 0.003 / 0.051 ± 0.003	Y = O (-18%)	0.026 / 0.021	Y = O (-19%)	MPS increased from postabsorptive values in both Y and O

								4h					
Katsanos et al. (2006)	NR-PGD, AGR	Leucine 26%<br>10 (3) / 8 (4)<br>Leucine 41%<br>10 (5) / 8 (4)	Leucine 26%<br>67 ± 2 / 31 ± 2<br>Leucine 41%<br>67 ± 2 / 29 ± 3	Healthy, physical activity level NA/no regular exercise	Fasted	6.7g EAA with either 26% or 41% leucine enrichment, orally	L-[ring-$^2H_5$]-phenyl-alanine, mixed, plasma	Infusion bolus (0h; 2.0 µmol/kg) and rate (0.05 µmol/kg/min)<br>Protein supple. (4h)<br>Biopsy (4h and 9h)<br>MPS duration (5h)	Leucine 26%<br>0.048 ± 0.006 / 0.063 ± 0.006<br>Leucine 41%<br>0.057 ± 0.008/ 0.058 ± 0.007	Leucine 26%<br>Y = O (31%)<br>Leucine 41%<br>Y = O (2%)	Leucine 26%<br>0.005 / 0.015<br>Leucine 41%<br>0.019 / 0.022	Leucine 26%<br>Y = O (200%)<br>Leucine 41%<br>Y = O (16%)	MPS increased only in Y with 26% leucine<br>MPS increased similarly in both Y and O with 41% leucine
Kiskini et al. (2013)	NR-PGD	12 (0) / 12 (0)	75 ± 1 / 21 ± 1	Healthy, physical activity level NA/no regular exercise	Fasted	20g intrinsically labeled casein, orally<br>60g carbohyd., orally	L-[ring-$^{13}C_6$]-phenyl-alanine ingested orally, mixed, plasma	Tracer enrichment in 20g casein: 37.4 mole percent excess (MPE)<br>Protein supple. (0h)<br>Biopsy (0h and 6h)<br>MPS duration 6h	0.044 ± 0.005 / 0.048 ± 0.003	Y = O (9%)	0.044 / 0.048	Y = O (9%)	MPS values were similar in both Y and O
Koopman et al. (2009)	NR-PGD	10 (0) / 10 (0)	64 ± 1 / 23 ± 1	Healthy, physical activity level NA/no regular exercise	Fasted	35g intrinsically labeled casein, orally	L-[ring-$^2H_5$]-phenyl-alanine & L-[1-^{13}C]-leucine, mixed, plasma	Infusion bolus (0h; 2.0 µmol/kg) and rate (0.046 µmol/kg/min)<br>Protein supple. (2h)<br>Biopsy (2h and 8h)<br>MPS duration 6h	Phenylalanine<br>0.063 ± 0.006 / 0.054 ± 0.004	Phenylalanine<br>Y = O (-14%)	Phenylalanine<br>0.063 /0.054	Phenylalanine<br>Y = O (-14%)	MPS values were similar in both Y and O with both tracers

Mitchell et al. (2017)	NR-PGD, (NR-SG)	8 (0) / 8 (0)	70 ± 1 / 20 ± 1	Healthy, physical activity level NA	Fasted	15g EAA, orally<br>Older men were allocated to EAA only (OLD)	L-[ring-$^{13}C_6$]-phenyl-alanine, myo, IC	Infusion bolus (0h; 0.3 mg/kg) and rate (0.6 mg/kg/h)<br>Protein supple. (3h)<br>Biopsy (3h, 4.5h and 7h)<br>MPS duration 1.5h and 4h	0-1.5h<br>0.034 ± 0.009 / 0.059 ± 0.010<br>1.5-3h<br>0.084 ± 0.005 / 0.095 ± 0.006<br>3-4h<br>0.054 ± 0.012 / 0.057 ± 0.008<br>Total synthesis (4h)<br>0.229 ± 0.023 / 0.286 ± 0.025	0-1.5h<br>Y = OLD (70%)<br>1.5-3h<br>Y = OLD (13%)<br>3-4h<br>Y = OLD (6%)<br>Total synthesis (4h)<br>Y > OLD (25%)*	0-1.5h<br>-0.017 / 0.003<br>1.5-3h<br>0.033 / 0.041<br>3-4h<br>0.003 / 0.003	0-1.5h<br>Y = OLD (120%)<br>1.5-3h<br>Y = OLD (24%)<br>3-4h<br>Y = OLD (17%)	MPS increased from postabsorptive values in both Y and O after 1.5h<br>Total MPS was greater in Y vs. O in both conditions
Paddon-Jones et al. (2004)	NR-PGD	7 (4) / 6 (4)	67 ± 1 / 34 ± 2	Healthy, recreationally active/no regular exercise	Fasted	15g EAA, orally	L-[ring-$^{2}H_5$]-phenyl-alanine, mixed, IC	Infusion bolus (0h; 2.0 µmol/kg) and rate (0.05 µmol/kg/min)<br>Protein supple. (5h)<br>Biopsy (5h and 9h)<br>MPS duration 4h	0.088 ± 0.011 / 0.103 ± 0.088	Y = O (17%)	0.032 / 0.039	Y = O (22%)	MPS increased from postabsorptive values in both Y and O
Pennings et al. (2011)	NR-PGD	12 (0) / 12 (0)	75 ± 1 / 21 ± 1	Healthy, physical activity level NA/no regular exercise	Fasted	20g casein, orally	L-[ring-$^{2}H_5$]-phenyl-alanine, mixed, plasma	Infusion bolus (0h; 2.0 µmol/kg) and rate (0.044 µmol/kg/min)<br>Protein supple. (2h)<br>Biopsy (2h and 8h)<br>MPS duration (6h)	0.058 ± 0.004 / 0.062 ± 0.004	Y = O (8%)	NA	NA	MPS values were similar in both Y and O

Symons et al. (2009)	NR-PGD, AGR	17 (7) / 17 (9)	68 ± 1 / 35 ± 1	Healthy, recreationally active/no regular exercise	Fasted	Lean ground beef, 26.5% protein, orally<br>Each age-group randomized to<br>30g protein n=10 (5) / 10(5)<br>90g protein n=7 (2) / 7 (4)	L-[ring-$^{13}C_6$]-phenyl-alanine, mixed, IC	Infusion bolus (0h; 2.0 µmol/kg) and rate (0.08 µmol/kg/min)<br>Protein supple. (6h)<br>Biopsy (6h and 11h)<br>MPS duration (5h)	30g pro 0.120 ± 0.006 / 0.126 ± 0.006<br>90g protein 0.120 ± 0.015 / 0.118 ± 0.013	30g pro Y = O (5%)<br>90g pro Y = O (-2%)	30g pro 0.040 / 0.045<br>90g pro 0.034 / 0.036	30g pro Y = O (13%)<br>90g pro Y = O (6%)	MPS increased from postabsorptive values in both Y and O with both 30g and 90g protein
Volpi et al. (1999)	NR-PGD	8 (2) / 7 (3)	71 ± 2 / 30 ± 2	Healthy, recreationally active/no regular exercise	Fasted	40g of AA dissolved in 530 ml drinks, given in boluses of 30 ml every 10 min, orally	L-[ring-$^2H_5$]-phenyl-alanine, mixed, IC	Infusion bolus (0h; 2.0 µmol/kg) and rate (0.08 µmol/kg/min)<br>Protein supple. (5h - 8h)<br>Biopsy (5h and 8h)<br>MPS duration (3h)	0.095 ± 0.014 / 0.079 ± 0.009	Y = O (-17%)	0.009 / -0.003	Y = O (-129%)	MPS increased from postabsorptive values in both Y and O
Volpi et al. (2000)	NR-PGD	5 (1) / 5 (3)	72 ± 1 / 30 ± 3	Healthy, recreationally active/no regular exercise	Fasted	40g of AA and 40g of glucose dissolved in 530 ml drinks, given in boluses of 30 ml every 10 min, orally	L-[ring-$^2H_5$]-phenyl-alanine, mixed, IC	Infusion bolus (0h; 2.0 µmol/kg) and rate (0.08 µmol/kg/min)<br>Protein supple. (5-8h)<br>Biopsy (5h and 8h)<br>MPS duration (3h)	0.048 ± 0.015 / 0.086 ± 0.012	Y > O (80%)*	-0.002 / 0.042	Y > O (-2321)*	MPS increased from postabsorptive values only in Y
Welle et al. (1994)	NR-PGD	7 (4) / 9 (5)	68 ± 1 / 23 ± 1	Healthy, physical activity level NA	Fasted	Liquid meals<br>~25g protein<br>~25g fat<br>~100g CHO	[1-^{13}C]-leucine, myo, plasma	Infusion bolus (0h; 1.5 mmol) and rate (1.5 mmol/h)	0.063 ± 0.004 / 0.088 ± 0.003	Y > O (40%)*	NA	NA	MPS increased from postabsorptive values in both Y and O, but more so in Y

						Given every 30min for 4h, orally		Protein supple. (-1h-4h)<br>Biopsy (1h and 4h)<br>MPS duration (3h)					

Table S2 - Schematic overview of studies involving post-prandial muscle protein synthesis

Study design: Non-randomized parallel group design (NR-PGD), age-group randomization (AGR), non-randomized sub-groups (NR-SG). Nutritional protocol: type / dose / administration. Total amount of the given dose was calculated when possible. Method for MPS: Type of tracer / type of MPS subfraction / type of precursor pool. Protocol for MPS assessment: Infusion details / intervention timing / muscle biopsy timing / MPS duration (timing relative to infusion initiation). MPS response: Absolute post-intervention scores, change-scores from post-absorptive scores, when possible, unit: %/hrs. Group difference: A: %-difference from absolute post-intervention scores, B: %-difference from change-scores, direction (%-difference relative to old), * denotes P < 0.05 as reported in the given study). All data are means ± SE and order-listed as old / young. AA = amino acids, AUC = area under curve, EAA = essential amino acids, E% = energy percentage, FFM = fat free mass, IC = intracellular, MPS = muscle protein synthesis, Myo = myofibrillar, NEAA = non-essential amino acids, O = old, Y = young, 1RM = one repetition maximum,
