# Supplementary material for: Age-related anabolic resistance and post-absorptive muscle protein synthesis: integrative evidence from a systematic review and meta-analysis
Source: Front Physiol. 2026 Jun 5;17:1740284. doi: 10.3389/fphys.2026.1740284 (PMC13278896; doi:10.3389/fphys.2026.1740284)
Supplement: Supplementary file 5 [file Table3.pdf]

| Reference | Study design | Sample size, n (n, females) | Age (yrs) | Habitual condition | Condition (fasted /fed) | Exercise | Protocol details | Method for MPS | Protocol for MPS assessment | MPS response (absolute) | Group difference (A) | MPS response (Δ) | Group difference (B) | Notes / Additional outcomes |
|---|---|---|---|---|---|---|---|---|---|---|---|---|---|---|
| Brook et al. (2016) | NR-PGD | 10 (0) / 10 (0) | 69 ± 1 / 23 ± 1 | Healthy, recreationally active/no RT | Fasted | Unilateral KE | 75% 1RM<br>6x8<br>Interset pause NA, | $D_2O$, myofibrillar, deuterium labeled alanine | Bolus (0h; 150 ml) and rate (50 ml/week)<br>Exercise (~0h)<br>Biopsy (~75min)<br>MPS duration ~75min | 0-75 min 1.356 ± 0.082 / 1.344 ± 0.092 | Y = O (-1%) | NA | NA | Data only from acute part of the study<br>MPS values were similar in both Y and O<br>Phosphorylation of P70S6K increased only in Y<br>Similar phosphorylation of mTOR, rpS6 and 4E-BP1 in Y and O |
| Fry et al. (2011) | NR-PGD | 16 (8) / 16 (8) | 70 ± 2 / 27 ± 2 | Healthy, recreationally active/no regular exercise | Fasted | Unilateral KE | 1 set at 45% and 8 sets at 70% 1RM<br>10 reps<br>3 min inter-set pause | L-[ring-$^{13}C_6$]-phenyl-alanine, mixed, IC | Infusion bolus (0h; 2.0 µmol/kg) and rate (0.05 µmol/kg/min)<br>Exercise (5h)<br>Biopsy (5h, 8h, 11h and 29h)<br>MPS duration 3h, 6h and 24h | 0-3h 0.059 ± 0.008 / 0.067 ± 0.003<br>0-6h 0.065 ± 0.008 / 0.077 ± 0.005<br>0-24h 0.063 ± 0.009 / 0.079 ± 0.008 | 0-3h Y > O (14%)*<br>0-6h Y > O (19%)*<br>0-24h Y > O (25%)* | 0-3h 0.005 / 0.015<br>0-6h 0.011 / 0.025<br>0-24h 0.009 / 0.027 | 0-3h Y > O (200%)*<br>0-6h Y > O (127%)*<br>0-24h Y > O (200%)* | MPS increased from post-absorptive values in both Y and O at all time points, but more so in Y<br>Greater phosphorylation of p70S6K1, 4E-BP1 (6h & 24h) and mTOR (24h) in Y compared to O<br>Tendency towards greater rpS6 phosphorylation in Y compared to O (p = 0.08)<br>Phosphorylation of ERK1/2 increased only in Y (6h & 24h) |
| Kumar et al. (2009) | NR-PGD, AGR | 25 (0) / 25 (0) | 70 ± 5 / 24 ± 6 | Healthy, recreationally active | Fasted | Unilateral isotonic KE and KF | 20% 1RM (n= 5 / 5) 3x27<br>40% 1RM (n= 5 / 5) 3x14 | [1,2-$^{13}C_2$]-leucine, myo, plasma | Infusion bolus (0h; 0.7 mg/kg) and rate (1 | 60-90% RM (pooled, n= 15/15)<br>0-1h | 60-90% RM<br>0-1h post RE Y = O (19%) | NA | NA | Overall MPS response (AUC) for 60-90% was higher in Y vs. O at 1-2h<br>Combined MPS |

| | | | | | | | | | | | | | | |
|---|---|---|---|---|---|---|---|---|---|---|---|---|---|---|
| | | | | | | Volume matched between intensities | 60% 1RM (n= 5 / 5) 3x9<br>75% 1RM (n= 5 / 5) 3x8<br>90% 1RM (n= 5 / 5) 6x3<br>2 min interset pause | | mg/kg/h)<br>Exercise (3h)<br>Biopsy (3h, 4h, 5h and 7h)<br>MPS duration post exercise 1h (0-1h, 1-2h) and 2h (2-4h) | 0.047 ± 0.017 / 0.056 ± 0.008<br>1-2h 0.073 ± 0.012 / 0.106 ± 0.01<br>2-4h 0.046 ± 0.014 / 0.052 ± 0.009<br>1-2h for 20% RM 0.04 ± 0.005 / 0.058 ± 0.023<br>40% RM 0.049 ± 0.008 / 0.067 ± 0.018<br>60% RM 0.071 ± 0.012 / 0.095 ± 0.003<br>75% RM 0.07 ± 0.012 / 0.108 ± 0.021<br>90%RM 0.069 ± 0.01 / 0.094 ± 0.014 | 1-2h post RE Y > O (45%)*<br>2-4h post RE Y = O (13%)<br>20-90% RM (combined) Y > O* | | | responses for all intensities at 3-4h was higher in Y vs. O<br>Greater phosphorylation of p70S6K1 and 4E-BP1 in Y vs. O 1h post RE |
| Kumar et al. (2012) | NR-PGD, AGR, CO | 12 (0) / 12 (0) | 70 ± 5 / 24 ± 6 | Healthy, recreationally active | Fasted | Unilateral isotonic KE and KF | 40% 1RM (3x14, n=6)<br>40% 1RM (6x14, n=6)<br>75% 1RM (3x8, n=6))<br>75% 1RM (6x8, n=6)<br>3 min interset pause | $[1,2\text{-}^{13}C_2]$-leucine, myo, plasma | Infusion bolus (0h; 0.7mg/kg) and rate (1mg/kg/h)<br>Exercise (3h)<br>Biopsy (3h, 4h, 5h and 7h)<br>MPS duration post exercise 1h (0-1h, 1-2h) and 2h (2-4h) | 40%, 3x14 0.008 ± 0.013 / 0.066 ± 0.022<br>40%, 6x14 0.127 ± 0.030 / 0.060 ± 0.019<br>75%, 3x8 0.065 ± 0.008 / 0.089 ± 0.031<br>75%, 6x8 0.136 ± 0.020 0.145 ± 0.033 | 40%, 3 sets Y > O (767%)*<br>40%, 6 sets Y = O (-53%)<br>75%, 3 sets Y = O (38%)<br>75%, 6 sets Y = O (6%) | 40%, 3 sets -0.031 / 0.026<br>40%, 6 sets 0.084 / 0.012<br>75%, 3 sets 0.027 / 0.049<br>75%, 6 sets 0.099 / 0.102 | 40%, 3 sets Y > O (183%)*<br>40%, 6 sets Y = O (-85%)<br>75%, 3 sets Y = O (80%)<br>75%, 6 sets Y = O (4%) | Overall MPS response (AUC) was greater in Y vs. O only at 40%, 3 sets.<br>AUC was greater for 6 sets compared to 3 sets at both 40% and 75% only in Y.<br>Tendency to greater MPS in O vs. Y at 40%, 6 sets<br>Phosphorylation of p70S6K1 was greater at 6 set at 75% in both Y and O, but only in O at 40% (no between group |

| | | | | | | | | | | | | | | |
|---|---|---|---|---|---|---|---|---|---|---|---|---|---|---|
| | | | | | | | | | | | | | | comparisons) |
| Lamon et al. (2016) | NR-PGD, AGR, CO | 10 (0) / 10 (0) | 67 ± 1 / 24 ± 1 | Healthy, recreationally active/no resistance exercise | Fasted | KE | 60% 1RM<br>3 x 14 sets<br>2 min interset pause | [ring-$_{13}C_6$] L-phenyl-alanine, mixed, IC. | Infusion bolus (0h; 0.34 mg/kg) and rate (0.0085 mg/kg/min)<br>Exercise (2h)<br>Biopsy (2h)<br>MPS duration 2h | Post ex 0-2h<br>0.071 ± 0.017 / 0.073 ± 0.005 | Post ex 0-2h<br>Y = O (3%) | NA | NA | MPS values were similar in both Y and O |
| Mayhew et al. (2009) | NR-PGD | 6 (NA) / 8 (NA) | 64 ± 1 / 27 ± 1 | Healthy, physical activity level NA/no RT | Fasted | SQ, LP and KE | 8-12RM<br>3 x 8-12<br>1.5 min interset pause | L-[ring-$^2H_5$]-phenyl-alanine, mixed, IC | Infusion bolus (0h; 2 µmol/kg) and rate (0.05 µmol/kg/min)<br>Exercise (5h)<br>Biopsy (29h)<br>MPS duration 24h | Post ex 0-24h<br>0.065 ± 0.017 / 0.109 ± 0.014 | Post ex 0-24h<br>Y = O (68%) | Post ex 0-24h<br>0.013 / 0.054 | Post ex 0-24h<br>Y = O (313%) | MPS increased from postabsorptive values only in Y, and tended to be greater than O.<br>Phosphorylation of Akt and 4E-BP1 only increased in Y<br>Phosphorylation of rpS6 increased only in O<br>No increase in phosphorylation of p70S6K1 (auto-inhibitory domain) in Y and O |
| Michie et al. (2024) | NR-PGD | 50 (24) / 30 (15) | 71 ± 1 / 27 ± 1 | Healthy, physical activity level NA | Fasted | KE | 70% 1RM<br>10 x 8 sets<br>Interset pause NA | [$_{13}C_6$]-phenyl-alanine, mixed, plasma | Infusion bolus (0h; 1 mg/kg) and rate (1 mg/kg FFM/hour)<br>Exercise (4h)<br>Biopsy (7h)<br>MPS duration 3h | Post ex 0-3h<br>1,362 ± 0,785 / 1,323 ± 0,563 | Post ex 0-3h<br>Y = O (-3%) | NA | NA | MPS values were similar in both Y and O |

| | | | | | | | | | | | | | | |
|---|---|---|---|---|---|---|---|---|---|---|---|---|---|---|
| Sheffield-Moore et al. (2005) | NR-PGD | | 67 ± 2 / 27 ± 3 | Healthy, physical activity level NA/no regular exercise | Fasted | KE | 80% 1RM<br>6x8<br>Interset pause NA | L-[ring-$^{2}H_{5}$]-phenyl-alanine, mixed, IC | Infusion bolus (0h; 2 µmol/kg) and rate (0.05 µmol/kg/min)<br>Exercise (3h)<br>Biopsy (3h, 4h, 5h and 7h)<br>MPS duration 1h, 2h and 4h | Post ex 0-10 min<br>0.12 ± 0.018 / 0.072 ± 0.005<br>Post ex 0-1h<br>0.089 ± 0.013 / 0.091 ± 0.007<br>Post ex 0-3h<br>0.079 ± 0.014 / 0.102 ± 0.013 | Post ex 0-10 min<br>Y = O (-40%)<br>Post ex 0-1h<br>Y = O (2%)<br>Post ex 0-3h<br>Y = O (29%) | Post ex 0-10 min<br>0.044 / 0.004<br>Post ex 0-1h<br>0.013 / 0.019<br>Post ex 0-3h<br>0.003 / 0.03 | Post ex 0-10 min<br>Y = O (-100%)<br>Post ex 0-1h<br>Y = O (46%)<br>Post ex 0-3h<br>Y = O (900%) | MPS increased from postabsorptive values after 1h only in O, and after 3h only in Y |

**Table S3 - Schematic overview of studies involving post-resistance exercise muscle protein synthesis**

Study design: Non-randomized parallel group design (NR-PGD), age-group randomization (AGR), cross-over (CO). Exercise: Exercises used in the protocol. Protocol details: Intensity / sets x reps / inter-set pauses. Method for MPS: Type of tracer / type of MPS subfraction / type of precursor pool. Protocol for MPS assessment: Infusion details / intervention timing / muscle biopsy timing / MPS duration (timing relative to infusion initiation). MPS response: Absolute post-intervention scores, change-scores from postabsorptive scores, when possible, unit: %/hrs. Group difference: A: %-difference from absolute post-intervention scores, B: %-difference from change scores, direction (%-difference relative to old), * denotes $P < 0.05$ as reported in the given study. All data are means ± SE and order-listed as old / young. AUC = area under curve, IC = intracellular, KE = knee extensions, KF = knee flexions, LP = leg press, MPS = muscle protein synthesis, Myo = myofibrillar, O = old, Plasma = Plasma amino acid enrichment, RT = resistance training, SQ = squat, Y = young, 1RM = one repetition maximum.
