# Supplementary material for: Age-related anabolic resistance and post-absorptive muscle protein synthesis: integrative evidence from a systematic review and meta-analysis
Source: Front Physiol. 2026 Jun 5;17:1740284. doi: 10.3389/fphys.2026.1740284 (PMC13278896; doi:10.3389/fphys.2026.1740284)
Supplement: Supplementary file 6 [file Table4.pdf]

Reference	Study design	Sample size, n (n, females)	Age (yrs)	Habitual condition	Condition (fasted/fed)	Exercise & protocol	Protein/AA stimulus	Method for MPS	Protocol for MPS assessment	MPS response (absolute)	Group difference (A)	MPS response (Δ)	Group difference (B)	Notes / Additional outcomes
Atherton et al. (2017)	NR-PGD, AGR	18 (0) / 18 (0)	70 ± 5 / 24 ± 6	Healthy, physical activity level NA	Fasted	Unilateral isotonic KE and KF<br>70% 1RM<br>6x8<br>3 min interset pause	10g protein (8g Casein + 2g whey)<br>4.2g leucine or alanine<br>Orally	[1,2-$^{13}C_2$]-leucine, myo, plasma	Infusion bolus (0h; 0.7 mg/kg) and rate (1 mg/kg/h)<br>Protein suppl. (3h)<br>Exercise (2.5h)<br>Biopsy (3h, 5h and 7h)<br>MPS duration 2h and 4h	Leucine<br>Post-ex 1h<br>0.067 ± 0.010 / 0.055 ± 0.013<br>Post-ex 2h<br>0.096 ± 0.011 / 0.110 ± 0.009<br>Post-ex 4h<br>0.072 ± 0.010 / 0.052 ± 0.006<br>Post-ex AUC<br>0.138 ± 0.018 / 0.151 ± 0.016<br><br>Alanine<br>Post-ex 1h<br>0.050 ± 0.012 / 0.059 ± 0.009<br>Post-ex 2h<br>0.079 ± 0.003 / 0.082 ± 0.006<br>Post-ex 4h<br>0.065 ± 0.006 / 0.053 ± 0.008<br>Post-ex AUC<br>0.115 ± 0.017 / 0.125 ± 0.012	Leucine<br>Post-ex 1h<br>Y = O (-18%)<br>Post-ex 2h<br>Y = O (15%)<br>Post-ex 4h<br>Y = O (-28%)<br>Post-ex AUC<br>Y = O (9%)<br>Alanine<br>Post-ex 1h<br>Y = O (19%)<br>Post-ex 2h<br>Y = O (3%)<br>Post-ex 4h<br>Y = O (-18%)<br>Post-ex AUC<br>Y = O (9%)	Leucine<br>Post-ex 1h<br>0.028 / 0.011<br>Post-ex 2h<br>0.057 / 0.066<br>Post-ex 4h<br>0.033 / 0.008<br>Post-ex AUC<br>NA<br><br>Alanine<br>Post-ex 1h<br>0.011 / 0.021<br>Post-ex 2h<br>0.040 / 0.040<br>Post-ex 4h<br>0.026 / 0.012<br>Post-ex AUC<br>NA	Leucine<br>Post-ex 1h<br>Y = O (-51%)<br>Post-ex 2h<br>Y = O (21%)<br>Post-ex 4h<br>Y = O (-67%)<br>Post-ex AUC<br>NA<br><br>Alanine<br>Post-ex 1h<br>Y = O (93%)<br>Post-ex 2h<br>Y = O (8%)<br>Post-ex 4h<br>Y = O (-42%)<br>Post-ex AUC<br>NA	In all age groups and subgroups, MPS was greater with leucine compared to alanine<br>Leucine enhanced p70S6K1 phosphorylation in O but not Y
Drummond et al. (2008)	NR-PGD	6 (0) / 7 (0)	70 ± 2 / 30 ± 2	Healthy, physical activity level NA / no regular	Fasted	Bilateral KE<br>70% 1RM<br>8x10<br>3 min	20g EAA<br>Orally	L-[ring-$^2H_5$]-phenyl-alanine, mixed, IC	Infusion bolus (0h; 2 µmol/kg) and rate (0.05	Post-ex +1h<br>0.045 ± 0.010 / 0.033 ± 0.012<br>Post-ex 1-3h<br>0.053 ± 0.011 /	Post-ex +1h<br>Y = O (-25%)<br>Post-ex 1-3h<br>Y > O (102%)*<br>Post-ex 3-6h	Post-ex +1h<br>-0.001 / -0.011<br>Post-ex 1-3h<br>0.007 /	Post-ex +1h<br>Y = O (647%)<br>Post-ex 1-3h<br>Y > O (765%)*<br>Post-ex 3-6h	MPS was greater in Y vs. O at 2h, but similar at 3h<br>Phosphorylation of Akt increased only in Y

				exercise		interset pause			µmol/kg/min) Protein suppl. (4h) Exercise (3h) Biopsy 4h, 6h and h) MPS duration 2h and 3h	0.108 ± 0.020 Post-ex 3-6h 0.161 ± 0.036 / 0.102 ± 0.007 Post-ex AUC 0.126 ± 0.028 / 0.105 ± 0.009	Y = O (-37%) Post-ex AUC Y = O (-17%)	0.064 Post-ex 3-6h 0.115 / 0.058 Post-ex AUC NA	Y = O (-50%) Post-ex AUC NA	Phosphorylation of mTOR, p70S6K and 4E-BP1 increased similarly in Y and O
Hermans et al. (2023)	NR-PGD	15 (0) / 14 (0)	73 ± 1 / 25 ± 1	Healthy, physical activity level NA / no to a low volume of regular exercise and no RT	Fasted	LP and KE 80% 1RM 4x8-10	30g protein (quark, dairy) Orally	L-[ring-$^{13}C_6$]-phenyl-alanine, mixed, plasma	Infusion bolus (0h; 2.25µmol/kg) and rate (0.05µmol/kg/min) Protein suppl. (3.5h) Exercise (2,5h) Biopsy (3.5h and 7.5h) MPS duration 4h	Post-ex 1-5h 0.078 ± 0.005 / 0.071 ± 0.006	Post-ex 1-5h Y = O (-9%)	Post-ex 1-5h 0.042 / 0.041	Post-ex 1-5h Y = O (-2%)	MPS increased from postabsorptive values in both Y and O
Horwath et al. (2024)	NR-PGD	10 (0) / 10 (0)	70 ± 1 / 22 ± 1	Healthy, recreationally active / no RT	Fasted	Unilateral KE 10RM 10x10 (first three as warm up at 30%, 50% and 70% of 10RM) 2 min interset pause	EAA drink containing 240mg EAA/bw (17.6g) Orally	L-[ring-$^{13}C_6$]-phenyl-alanine, myo, plasma & IC	Infusion bolus (0h; 2 µmol/kg) and rate (0.05 µmol/kg/min) Protein suppl. (3h,15min) Exercise (2,5h) Biopsy (3h 15min,	IC Post-ex 0-1h 0.106 ± 0.010 / 0.103 ± 0.015 Post-ex 0-3h 0.119 ± 0.007 / 0.120 ± 0.008	IC Post-ex 0-1h Y = O (-4%) Post-ex 0-3h Y = O (1%)	IC Post-ex 0-1h 0.057 / 0.046 Post-ex 0-3h 0.069 / 0.062	IC Post-ex 0-1h Y = O (-21%) Post-ex 0-3h Y = O (-10%)	MPS increased from post-absorptive values in both Y and O at all time points and both precursor pools Similar phosphorylation of mTOR, p70S6K and rpS6 in Y and O Greater phosphorylation of 4E-BP1 in O vs. Y

									4h15min and 6h15min)<br>MPS duration 1h and 3h					
Koopman et al. (2006)	NR-PGD, AGR, CO	8 (0) / 8 (0)	76 ± 2 / 20 ± 1	Healthy, physical activity level NA/no regular exercise	Fasted	LP and KE<br>40-75% 1RM<br>6x10<br>2 sets at 40, 55 and 75%<br>2 min interset pause	Boluses (1.33 mL/kg) of mixed drink, every 30 min for 5.5h, orally<br>~200g CHO<br>~66g whey protein<br>3<br>~12.3 leucine	L-[ring-13C6]-phenyl-alanine, mixed, plasma	Infusion bolus (0h; 2 µmol/kg) and rate (0.049 µmol /kg/min)<br>Protein suppl. (~2h - 7.5h)<br>Exercise (1h)<br>Biopsy (~2h and 8h)<br>MPS duration 6h	Post-ex 0-6h<br>0.072 ± 0.006 / 0.082 ± 0.005	Post-ex 0-6h<br>Y > O (14%)*	NA	NA	MPS increased from postabsorptive values in both Y and O, but more so in Y
Lalia et al. (2017)	NR-PGD	12 (7) / 12 (6)	76 ± 1 / 27 ± 1	Healthy, physical activity level NA/no to a low volume of regular exercise	Fed 4h prior to and 2h post RE	Unilateral KE<br>70%<br>8 x 10<br>3 min interset pause	35g protein<br>88g CHO<br>23g fat<br>Orally	L-[ring-$^{13}C_6$]-phenyl-alanine, my & mixed, IC	Infusion bolus (0h; 1.5/kg FFM) and rate (1.5 mg/kg FFM/h)<br>Protein suppl. (7h and 13h)<br>Exercise (11h)<br>Biopsy (26h, 29h)<br>MPS duration 3h	Mixed<br>Post-ex 15-18h<br>0.100 ± 0.017 / 0.090 ± 0.006<br>Myo<br>Post-ex 15-18h<br>0.089 ± 0.012 / 0.095 ± 0.010	Mixed<br>Post-ex 15-18h<br>Y = O (-10%)<br>Myo<br>Post-ex 15-18h<br>Y = O (7%)	Mixed<br>Post-ex 15-18h<br>0.028 / 0.023<br>Myo<br>Post-ex 15-18h<br>0.017 / 0.022	Mixed<br>Post-ex 15-18h<br>Y = O (28%)<br>Myo<br>Post-ex 15-18h<br>Y = O (29%)	MPS increased from postabsorptive values only in Y in both muscle fractions
Pennings et al. (2011)	NR-PGD	12 (0) / 12 (0)	73 ± 1 / 21 ± 1	Healthy, physical activity level	Fasted	LP and KE<br>40-75% 1RM<br>6x10	20g casein<br>Orally	L-[ring-$^{2}H_5$]-phenyl-alanine, mixed,	Infusion bolus (0h; 2 µmol/kg) and rate	Post-ex 0-6h<br>0.074 ± 0.003 / 0.076 ± 0.003	Post-ex 0-6h<br>Y = O (3%)	NA	NA	MPS values were similar in both Y and O

				NA/no regular exercise		2 sets at 40, 55 and 75%		plasma	(0.044 µmol /kg/min)<br>Protein suppl. (2h)<br>Exercise (1.5h)<br>Biopsy (2h and 8h)<br>MPS duration 6h					
Phillips et al. (2017)	NR-PGD	17 (~50/50) / 11 (~50/50)	70 ± 3 / 24 ± 1	Healthy, physical activity level NA/no regular exercise	Fasted	KE<br>75% 1RM<br>6x8<br>Interset pause NA	Liquid feed of normal mixed meal<br>23g Protein<br>72g CHO<br>23g fat	[1,2-$^{13}C_2$]-leucine, myo, plasma	Infusion bolus (0h; 0.66 mg/kg) and rate (1 mg/kg/min, increased to 1.2 mg/kg/min when feed was given)<br>Protein suppl. (~2.5h-5h)<br>Exercise (2h)<br>Biopsy (~2.5h and 5h)<br>MPS duration ~2.5h	Post-ex 0-2.5h 0.083 ± 0.003 / 0.081 ± 0.008	Post-ex 0-2.5h Y = O (-2%)	Post-ex 0-2.5h 0.041 / 0.037	Post-ex 0-2.5h Y = O (-10%)	MPS values increased similarly from postabsorptive values in both Y and O<br>Only data from the acute part of the study included
Symons et al. (2011)	NR-PGD	7 (4) / 7 (4)	67 ± 2 / 29 ± 3	Healthy, recreationally active/no regular exercise	Fasted	Isotonic KE<br>80% 1RM<br>8x6<br>2min interset pause	340g serving of lean ground beef, orally 660kcal 90g protein 33g fat	L-[ring-$^{13}C_6$]-phenyl-alanine, mixed, IC	Infusion bolus (0h; 2µmol/kg) and rate (0.08µmol/kg/min)<br>Protein suppl. (~0.5h)	Post-ex 0-5h 0.152 ± 0.017 / 0.156 ± 0.021	Post-ex 0-5h Y = O (3%)	NA	NA	MPS values increased similarly from post-absorptive values in both Y and O

									Exercise (5h)<br>Biopsy (5h and 10h)<br>MPS duration 5h					

Table S4 - Schematic overview of studies involving post-prandial and post-resistance exercise muscle protein synthesis

Study design: Non-randomized parallel group design (NR-PGD), age-group randomization (AGR), cross-over (CO). Nutritional protocol: type / dose / administration. Total amount of the given dose was calculated when possible. Exercise & protocol: exercise(s) used, protocol (intensity / sets x reps / inter-set pauses). Method for MPS: Type of tracer / type of MPS subfraction / type of precursor pool. Protocol for MPS assessment: Infusion details / intervention timing / muscle biopsy timing / MPS duration (timing relative to infusion initiation). MPS response: Absolute post-intervention scores, change-scores from post-absorptive scores, unit: %/hrs. Group difference: A: %-difference from absolute post-intervention scores, B: %-difference from change scores, direction (%-difference relative to old), * denotes $P < 0.05$ as reported in the given study. All data are means ± SE and order listed as old / young. IC = intracellular, KE = knee extensions, KF = knee flexions, LP = leg press, MPS = muscle protein synthesis, Myo = myofibrillar, O = old, Post-ex = post exercise, Y = young, 1RM = one repetition maximum.
