# Supplementary material for: Age-related anabolic resistance and post-absorptive muscle protein synthesis: integrative evidence from a systematic review and meta-analysis
Source: Front Physiol. 2026 Jun 5;17:1740284. doi: 10.3389/fphys.2026.1740284 (PMC13278896; doi:10.3389/fphys.2026.1740284)
Supplement: Supplementary file 7 [file Table5.pdf]

Reference	Study design	Sample size, n (n, females)	Age (yrs)	Habitual condition	Condition (fasted/fed)	Exercise & protocol	Protein/AA stimulus	Method for MB	MPB response	Group difference	Notes / Additional outcomes
**Protein studies (n=10)**											
Chevalier et al. (2011)	NR-PGD	8 (8) / 8 (8)	73 ± 3 / 24 ± 1	Healthy, low physical activity level	Fasted	-	Infusion clamps: Hyperinsulinemic (0,4 mU/kg LBM/min) Hyperglycemic (8 mmol/L) Hyperaminoacidemic (700 µmol/L BCAA)	Leucine Ra, 1-pool model (mod. Steele)	1.8 ± 0.1 / 1.7 ± 0.1 µmol/kg LBM/min	Y < O (-6%)*	Ra was less suppressed in O vs. Y
Dillon et al. (2011)	NR-PGD	7 (4) / 7 (5)	67 ± 2 / 30 ± 2	Healthy, physical activity level NA/no regular exercise	Fasted	-	AA infusion EAA and NEAA (0.45 ml/kg (prime), 1.35 ml/kg/h (continuous, 3h)) Pharmacological vasodilation	Phenyl-alanine kinetics, 3-pool model	78 ± 13 / 99 ± 16 nmol/min/100ml leg vol	Y = O (28%)	F_{MO} increased similarly in Y and O
Gorissen et al. (2014)	NR-PGD, AGR	13 (0) / 12 (0)	76 ± 1 / 20 ± 1	Healthy, physical activity level NA/no regular exercise	Fasted	-	20g Casein (1.4g leucin), orally 60g carbohydrate, orally	Phenyl-alanine Ra, 1-pool model (mod. Steele)	Pro 0.434 ± 0.013 / 0.574 ± 0.014 Pro + CHO 0.479 ± 0.015 / 0.575 ± 0.030 (µmol/kg/min)	Pro Y = O (-24%) Pro + CHO Y = O (17%)	Ra increased similarly in Y and O

Groen et al (2016)	NR-PGD	24 (0) / 24 (0)	68 ± 1 / 22 ± 1	Healthy, physical activity level NA	Fasted	-	20g Casein (30mg labeled phenyl-alanine), orally<br>Insulin (0.30 mU/min/100 mL leg volume), intravenously	Phenyl-alanine Ra, 1-pool model (mod. Steele)	CON 127.7 ± 25.7 / 141.6 ± 58.3<br>Insulin treated 100 ± 52.7 / 208.3 ± 80.5<br>(nmol/kg/min)	CON Y = O (11%)<br>Insulin Y > O (108%)*	Ra was greater in Y-insulin compared to Y-CON and both O groups
Katsanos et al. (2006)	NR-PGD, AGR	Leucine 26%: 10 (3) / 8 (4)<br>Leucine 41%: 10 (5) / 8 (4)	Leucine 26%: 67 ± 2 / 31 ± 2<br>Leucine 41%: 67 ± 2 / 29 ± 3	Healthy, physical activity level NA/no regular exercise	Fasted	-	6.7g of EAA, orally	Phenyl-alanine kinetics, 1-pool model (mod. Steele)	26% leucine 41 ± 3 / 43 ± 4<br>41% leucine 42 ± 6 / 34 ± 6<br>nmol/min/100ml leg vol	26% leucine Y = O (2%)<br>41% leucine Y = O (-17%)	Ra values did not change after EAA in Y or O in any groups
Koopman et al. (2009)	NR-PGD	10 (0) / 10 (0)	64 ± 1 / 23 ± 1	Healthy, physical activity level NA/no regular exercise	Fasted	-	35g intrinsically labeled casein, orally	Phenyl-alanine Ra, one-pool model (mod. Steele)	0.41 ± 0.01 / 0.5 ± 0.01<br>(µmol/kg/min)	Y > O (22%)*	Ra was greater in Y vs. O
Paddon-Jones et al. (2004)	NR-PGD	7 (4) / 6 (4)	67 ± 1 / 34 ± 2	Healthy, recreationally active/no regular exercise	Fasted	-	15g EAA, orally	Phenyl-alanine Ra, 2-poolmodel	39.8 ± 6.6 / 40.7 ± 8.4<br>(nmol/min/100ml leg vol)	Y = O (2%)	Average Ra values did not change after EAA in Y or O
Pennings et al. (2011)	NR-PGD	12 (0) / 12 (0)	75 ± 1 / 21 ± 1	Healthy, physical activity level NA/no regular exercise	Fasted	-	20g casein	Phenyl-alanine Ra, 1-pool model (mod. Steele)	Cannot be obtained from graph	Y = O (%NA)	Ra increased similarly in both Y and O
Volpi et al. (1999)	NR-PGD	8 (2) / 7 (3)	71 ± 2 / 30 ± 2	Healthy, recreationally active/no regular exercise	Fasted	-	40g of AA dissolved in 530ml drinks, given in boluses of 30ml every 10min	Phenyl-alanine kinetics, 3-pool model	51 ± 11 / 59 ± 10<br>(nmol/min/100ml leg vol)	Y = O (16%)	F_{MO} increased similarly in Y and O

Volpi et al. (2000)	NR-PGD	5 (1) / 5 (3)	72 ± 1 / 30 ± 3	Healthy, recreationally active/no regular exercise	Fasted	-	40g of AA and 40g of glucose dissolved in 530ml drinks, given in boluses of 30ml every 10min	Phenyl-alanine kinetics, 3-pool model	41 ± 11 / 77 ± 10 (nmol/min/100ml leg vol)	Y = O (88%)	F_{MO} increased similarly in Y and O
**Resistance exercise studies (n=1)**											
Sheffield Moore et al. (2005)	NR-PGD	6 (0) / 6 (0)	67 ± 2 / 27 ± 3	Healthy, physical activity level NA/no regular exercise	Fasted	KE, 80% 1RM 6x8 Interset pause (NA)	-	Phenyl-alanine kinetics, 3-pool model	10 min post RE 156.85 ± 62 / 8.42 ± 20.2 (nmol/min/100ml leg vol)	Y = O (-95%)	F_{MO} increased only in O
**Combination of resistance exercise and protein (n=2)**											
Koopman et al. (2006)	NR-PGD, AGR, CO	8 (0) / 8 (0)	76 ± 2 / 20 ± 1	Healthy, physical activity level NA/no regular exercise	Fasted	LP and KE 40-75% 1RM 10 x 6 2 min interset pause	Boluses (1.33mL/kg) of mixed drink, every 30 min for 5.5h, orally: ~200g CHO ~66g whey protein ~12.3 g leucine	Phenyl-alanine Ra, 2 -pool model	0.56 ± 1.41 / 11.27 ± 1.68 (µmol/kg/h)	Y > O (1912%)*	Ra was greater in Y compared to O
Pennings et al. (2011)	NR-PGD	12 (0) / 12 (0)	73 ± 1 / 21 ± 1	Healthy, physical activity level NA/no regular exercise	Fasted	LP and KE 40-75% 1RM 6x10 2 sets at 40, 55 and 75%	20g casein	Phenylalanin Ra, 1-pool model (Steele equation)	Cannot be obtained from graph	Y = O (%NA)	Ra increased similarly in both Y and O

Table S5 - Schematic overview of studies involving post-prandial and/or post-resistance exercise muscle protein breakdown

Table data represent protein breakdown; the majority of values are based on endogenous rate of tracer (leucine/phenyl alanine) appearance (based on the 1- or 3-pool), which serves as an indirect measure of muscular protein degradation. Study design: Non-randomized parallel group design (NR-PGD), age-group randomization (AGR), cross-over (CO). Nutritional protocol: type / dose / administration. Exercise & protocol: and exercise(s) used, protocol (intensity / sets x reps / inter-set pauses). Method for MPB: Estimated muscle protein degradation based on tracer kinetics (1-, 2-, or 3-pool models), see methods section for further details. MPB response: Absolute post-intervention MPB values. Group difference: %-difference from absolute post-intervention scores, direction (%-difference relative to old), * denotes P < 0.05 as reported in the given study. All data are means ± SE and order listed as old / young. F_{MO} = Rate of tracer outflow from protein breakdown, KE = knee extensions, KF = knee flexions, LP = leg press, MPB = muscle protein breakdown, O = old, Ra = Rate of appearance, Y = young, 1RM = one repetition maximum
