# Supplementary material for: Age-related anabolic resistance and post-absorptive muscle protein synthesis: integrative evidence from a systematic review and meta-analysis
Source: Front Physiol. 2026 Jun 5;17:1740284. doi: 10.3389/fphys.2026.1740284 (PMC13278896; doi:10.3389/fphys.2026.1740284)
Supplement: Supplementary file 8 [file Table6.pdf]

Reference	Study design	Sample size, n (n, females)	Age (yrs)	Habitual condition	Condition (fasted/fed)	Protein/AA stimulus	Timepoint for plasma AA	Plasma AA response	Group difference	Notes / Additional outcomes
**Protein studies (n=12)**										
Cuthbertson et al. (2005)	NR-PGD	24 (0) / 20 (0)	70 ± 1 / 28 ± 1	Healthy, physical activity level NA	Fasted	2.5g EAA<br>5g EAA<br>10g EAA<br>20g EAA<br>Hyperinsulinemic clamp 360 m IU·m^{-2} body surface area ·h^{-1}<br>Ocreotide 1.8 mg·kg^{-1}·h^{-1}	AUC during 2.5h postprandial period	Peak EAA (µmol/L)<br>957 ± 140 /<br>788 ± 30<br>Leucine (AUC) at 10g EAA<br>48643 ± 3216 /<br>20100 ± 6432<br>Leucine (AUC) at 20g EAA<br>74572 ± 7035 /<br>43015 ± 6633	Peak EAA<br>Y < O (-18%)*<br>Leucine at 10g EAA<br>Y < O (-599%)*<br>Leucine at 20g EAA<br>Y < O (-42%)*	Peak plasma EAA was greater in O vs. Y<br>Leucine AUC was greater in O vs. Y at 10g and 20g EAA
Gorissen et al. (2014)	NR-PGD, AGR	13 (0) / 12 (0)	76 ± 1 / 20 ± 1	Healthy, physical activity level NA/no regular exercise	Fasted	20g Casein, orally<br>60g carbohydrate, orally	2h post AA	Leucine (µmol/L)<br>Pro<br>221 ± 13 /<br>223 ± 10<br>Pro + CHO<br>142 ± 8 /<br>133 ± 11	Pro<br>Y = O (1%)<br>Pro + CHO<br>Y = O (-6%)	Trend for plasma leucine to be higher (p=0.10) in O, in both conditions
Groen et al. (2016)	NR-PGD	24 (0) / 24 (0)	68 ± 1 / 22 ± 1	Healthy, physical activity level NA	Fasted	20g Casein, orally<br>Insulin (0.30 mU/min/100 mL leg volume), intravenously	Peek AA reached between 20min and 40min post AA	Leucine (µmol/L)<br>Control<br>218 ± 25 /<br>210 ± 27<br>Insulin treated<br>175 ± 32 /	Control<br>Y = O (-4%)<br>Insulin treated<br>Y < O (-9%)*	When both groups combined, greater plasma leucine in O vs. Y

								160 ± 28		
Hermans et al. (2023)	NR-PGD	15 (0) / 14 (0)	73 ± 1 / 25 ± 1	Healthy, physical activity level NA/no to low regular exercise and no RT	Fasted	30g of protein from Quark (dairy product)	During 4h postprandial period	Leucine (iAUC, mmol/4h) 37 ± 5 / 34 ± 4 Total AA (iAUC, mmol/4h) 250 ± 35 / 218 ± 35	Leucine Y = O (10%) Total AA Y < O (15%)	Similar plasma leucine in Y and O Greater total AA in O vs. Y
Katsanos et al. (2006)	NR-PGD, AGR	Leucine 26%: 10 (3) / 8 (4) Leucine 41%: 10 (5) / 8 (4)	Leucine 26%: 67 ± 2 / 31 ± 2 Leucine 41%: 67 ± 2 / 29 ± 3	Healthy, physical activity level NA/no regular exercise	Fasted	Ingestion of 6.7g EAA with 26% or 41% leucine enrichment	NA	Leucine (nmol/L) 26% 442 ± 21 / 437 ± 2 41% 710 ± 52 / 583 ± 63	26% Y = O (-1%) 41% Y = O (-18%)	Similar plasma leucine in Y and O in both conditions
Kiskini et al. (2013)	NR-PGD	12 (0) / 12 (0)	75 ± 1 / 21 ± 1	Healthy, physical activity level NA/no regular exercise	Fasted	20g casein, orally 60g carbohydrate, orally	1.5h post AA	Leucine (µmol/L) 141 ± 7 / 175 ± 12 Total AA (µmol/L) 2568 ± 86 / 2929 ± 126	Leucine Y > O (24%)* Total AA Y = O (14%)	Greater plasma leucine in Y vs. O Similar total plasma AA in Y and O
Koopman et al. (2009)	NR-PGD	10 (0) / 10 (0)	64 ± 1 / 23 ± 1	Healthy, physical activity level NA/no regular exercise	Fasted	35g casein, orally	During 6h postprandial period	Leucine (AUC, mmol*6h/L) 5.3 ± 1.5 / 3.1 ± 0.7	Y < O (-42%)*	Greater plasma leucine in O vs. Y
Mitchell et al. (2017)	NR-PGD, NR-SG	8 (0) / 8 (0)	70 ± 0.8 / 19.7 ± 0.5	Healthy, physical activity level NA	Fasted	15g EAA, orally	NA	Leucine (µM) 720 ± 29 / 567 ± 31	Y < O (-21%)*	Greater plasma leucine in O vs. Y
Pennings et al. (2011)	NR-PGD	12 (0) / 12 (0)	75 ± 1 / 21 ± 1	Healthy, physical activity level NA/no regular exercise	Fasted	20g casein	NA	Cannot be obtained from graph	Y < O (%NA)*	Greater plasma leucine in O vs. Y

Volpi et al. (1999)	NR-PGD	8 (2) / 7 (3)	71 ± 2 / 30 ± 2	Healthy, recreationally active/no regular exercise	Fasted	40g of AA dissolved in 530ml drinks, given in boluses of 30ml every 10min	3h post AA	Leucine (µmol/L) 443 ± 23 / 438 ± 41	Y = O (-1%)	Similar plasma leucine in Y and O
Volpi et al. (2000)	NR-PGD	5 (1) / 5 (3)	72 ± 1 / 30 ± 3	Healthy, recreationally active/no regular exercise	Fasted	40g of AA and 40g of glucose dissolved in 530ml drinks, given in boluses of 30ml every 10min	NA	Leucine (µmol/L) 347 ± 46 / 452 ± 65	Y = O (30%)	Similar plasma leucine in Y and O
Welle et al. (1994)	NR-PGD	7 (4) / 9 (5)	68 ± 1 / 23 ± 1	Healthy, physical activity level NA	Fasted	Liquid meals<br>~25g protein<br>~25g fat<br>~100g CHO<br>Given every 30min for 4h, orally	NA	Leucine (µM) 176 ± 7 / 158 ± 17	Y = O (-10%)	Similar plasma leucine in Y and O

Table S6 - Schematic overview of studies involving post-prandial plasma amino acid concentration

Table data represents post-prandial plasma amino acid (AA) concentrations. Study design: Non-randomized parallel group design (NR-PGD), age-group randomization (AGR), non-randomized sub-groups (NR-SG). Protein/AA stimulus: type / dose / administration. Plasma AA: Absolute postprandial plasma amino acid concentrations. Group difference: %-difference from absolute postprandial plasma AA values, direction (%-difference relative to old), * denotes P < 0.05 as reported in the given study. All data are means ± SE and order-listed as old / young.
