# Supplementary material for: Age-related anabolic resistance and post-absorptive muscle protein synthesis: integrative evidence from a systematic review and meta-analysis
Source: Front Physiol. 2026 Jun 5;17:1740284. doi: 10.3389/fphys.2026.1740284 (PMC13278896; doi:10.3389/fphys.2026.1740284)
Supplement: Supplementary file 9 [file Table7.pdf]

Reference	Confounding bias	Participants selection bias	Intervention classification bias	Deviation from intended intervention bias	Missing data bias	Outcome measurement bias	Selection of the reported result bias	Overall rating of bias
Atherthon et al. (2017)	🟢	🟢	🟢	🟢	🟢	🟢	🟢	🟢
Babraj et al. (2005)	🟡	🟡	🟢	🟢	🟡	🟢	🟡	🟡
Balagopal et al. (1997)	🟢	🟢	🟢	🟢	🟡	🟢	🟡	🟡
Brook et al. (2016)	🟡	🟢	🟢	🟢	🟡	🟢	🟢	🟡
Chevalier et al. (2011)	🟡	🟡	🟢	🟢	🟢	🟢	🟡	🟡
Cuthbertson et al. (2005)	🟢	🟡	🟢	🟢	🟢	🟢	🟢	🟡
Dillon et al. (2011)	🟡	🟡	🟢	🟢	🟡	🟢	🟡	🟡
Drummond et al. (2008)	🟡	🟢	🟢	🟢	🟡	🟢	🟡	🟡

Durham et al. (2010)	🟡	🟢	🟢	🟢	🟡	🟢	🟡	🟡
Fry et al. (2011)	🟡	🟢	🟢	🟢	🟢	🟢	🟢	🟡
Gorissen et al. (2014)	🟢	🟢	🟢	🟢	🟢	🟢	🟢	🟢
Groen et al. (2016)	🟢	🟢	🟢	🟢	🟢	🟢	🟢	🟢
Guillet et al. (2004)	🟡	🟢	🟢	🟢	🟤	🟡	🟡	🟤
Hasten et al. (2000)	🟡	🟡	🟢	🟢	🟡	🟢	🟡	🟡
Henderson et al. (2009)	🟢	🟢	🟢	🟢	🟢	🟢	🟢	🟢
Hermans et al. (2023)	🟢	🟢	🟢	🟢	🟢	🟢	🟢	🟢
Horwath et al. (2024)	🟡	🟢	🟢	🟢	🟡	🟡	🟢	🟡

Katsanos et al. (2006)	🟡	🟢	🟢	🟢	🟢	🟢	🟢	🟡
Kiskini et al. (2013)	🟡	🟢	🟢	🟢	🟢	🟢	🟢	🟡
Koopman et al. (2006)	🟢	🟢	🟢	🟢	🟡	🟢	🟡	🟡
Koopman et al. (2009)	🟢	🟢	🟢	🟢	🟡	🟢	🟢	🟡
Kumar et al. (2009)	🟡	🟢	🟢	🟢	🟢	🟢	🟢	🟡
Kumar et al. (2012)	🟡	🟢	🟢	🟢	🟡	🟢	🟢	🟡
Laila et al. (2017)	🟢	🟢	🟢	🟢	🟡	🟢	🟢	🟡
Lamon et al. (2016)	🟡	🟢	🟢	🟢	🟢	🟢	🟢	🟡
Mayhew et al. (2009)	🟢	🟢	🟢	🟢	🟤	🟢	🟢	🟤

Mitchell et al. (2017)	🟢	🟢	🟢	🟢	🟡	🟢	🟢	🟡
Michie et al. (2024)	🟡	🟢	🟢	🟢	🟢	🟢	🟢	🟡
Paddon-Jones et al. (2004)	🟢	🟢	🟢	🟢	🟡	🟢	🟢	🟡
Pennings et al. (2011)	🟢	🟢	🟢	🟢	🟢	🟢	🟢	🟢
Phillips et al. (2017)	🟡	🟢	🟢	🟢	🟢	🟢	🟢	🟡
Rasmussen et al. (2006)	🟡	🟢	🟢	🟢	🟡	🟢	🟢	🟡
Rooyackers et al. (1996)	🟡	🟢	🟢	🟢	🟢	🟢	🟢	🟡
Sheffield-Moore et al. (2005)	🟢	🟢	🟢	🟢	🟡	🟢	🟢	🟡
Smeuninx et al. (2017)	🟡	🟡	🟢	🟢	🟢	🟢	🟢	🟡

Symons et al. (2009)	🟢	🟢	🟢	🟢	🟢	🟢	🟢	🟢
Symons et al. (2011)	🟢	🟢	🟢	🟢	🟡	🟢	🟢	🟡
Toth et al. (2005)	🟡	🟡	🟢	🟢	🟡	🟢	🟢	🟡
Volpi et al. (1999)	🟡	🟢	🟢	🟢	🟡	🟢	🟢	🟡
Volpi et al. (2000)	🟡	🟢	🟢	🟢	🟤	🟢	🟢	🟤
Volpi et al. (2001)	🟢	🟢	🟢	🟢	🟢	🟢	🟢	🟢
Walrand et al. (2008)	🟡	🟢	🟢	🟢	🟡	🟢	🟢	🟡
Welle et al. (1993)	🟤	🟢	🟢	🟢	🟡	🟢	🟢	🟤
Welle et al. (1994)	🟡	🟢	🟢	🟢	🟡	🟢	🟢	🟡

Welle et al. (1995)	● (brown)	● (green)	● (green)	● (green)	● (yellow)	● (green)	● (green)	● (brown)
Yarasheki et al. (1993)	● (yellow)	● (green)	● (green)	● (green)	● (brown)	● (green)	● (green)	● (brown)

Table S7 - Schematic overview of Risk of Bias

Risk of Bias assessment using the “Risk Of Bias In Non-randomized Studies of Interventions (ROBINS-I). ROBINS-I evaluate internal validity within the domains: ‘Confounding Bias’, ‘Participant Selection Bias’, ‘Intervention Classification Bias’, ‘Deviation from the Intended Intervention’, ‘Missing Data’, ‘Missing Outcomes’, and ‘Selection of Reported Results’. Green circles represent “Low RoB”, yellow circles “Moderate RoB”, and brown circles “Serious Risk of Bias”.
